# Supplementary material for: Temperature-dependent regulation of upstream open reading frame translation in S. cerevisiae
Source: BMC Biol. 2019 Dec 6;17:101. doi: 10.1186/s12915-019-0718-5 (PMC6898956; doi:10.1186/s12915-019-0718-5)
Supplement: Supplementary file 1 — Additional file 1: Figure S1. β-galactosidase activity assay to calculate usage of NCC start site at multiple growth temperature. Figure S2. Temperature dependent alterations in usage of NCC start site are not evoked due to changes in the levels of initiation factors. Figure S3. Ribosome profiling at multiple growth temperatures. Figure S4. Changes in growth temperature lead to changes in translation of uORFs. Figure S5. Analysis of translation efficiency changes of conserved uORFs upon changes in growth temperature. Figure S6. Analysis of the properties of various sets of uORFs and the mRNAs harboring them. Figure S7. Analysis of uORFs showing highest changes in translation at 20 °C and 37 °C. Figure S8. Boxplot analyses of 5’-UTR features for the most highly regulated NCC uORFs. Figure S9. Analysis of TE-changes of mORFs. Figure S10. Wiggle track images of examples of mRNAs that show temperature-dependent changes in translation of one or more uORF and the mORF. Figure S11. uORF-mediated temperature-dependent regulation of AGA1 mRNA. [file 12915_2019_718_MOESM1_ESM.pdf]

Additional file 1: Supplementary Figures 1-11 and legends.

Supplementary Figures and legends:

Figure S1) β-galactosidase activity assay to calculate usage of NCC start site at multiple growth temperature.

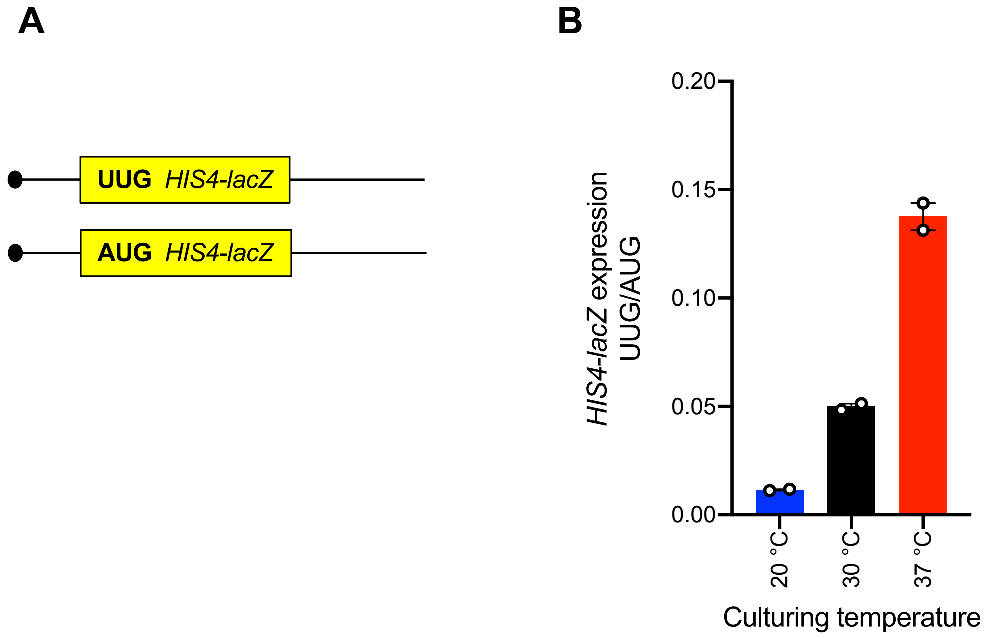

(A) Schematic of *HIS4-lacZ* reporters used in this study. **(B)** BY4741 cells with *HIS4-lacZ* reporters with AUG or UUG start codons were cultured at 20 °C, 30 °C or 37 °C to an A_{600} of 0.6 to 0.8 and β-galactosidase specific activity was measured in total cell extracts in units of ONPG cleaved per minute per mg of total protein in the extract. Ratios of expression from the UUG reporter to the AUG reporter were calculated and plotted as averages from two independent biological replicates (shown in open circles) with average deviation as error bars.

Figure S2) Temperature dependent alterations in usage of NCC start site are not evoked due to changes in the levels of initiation factors.

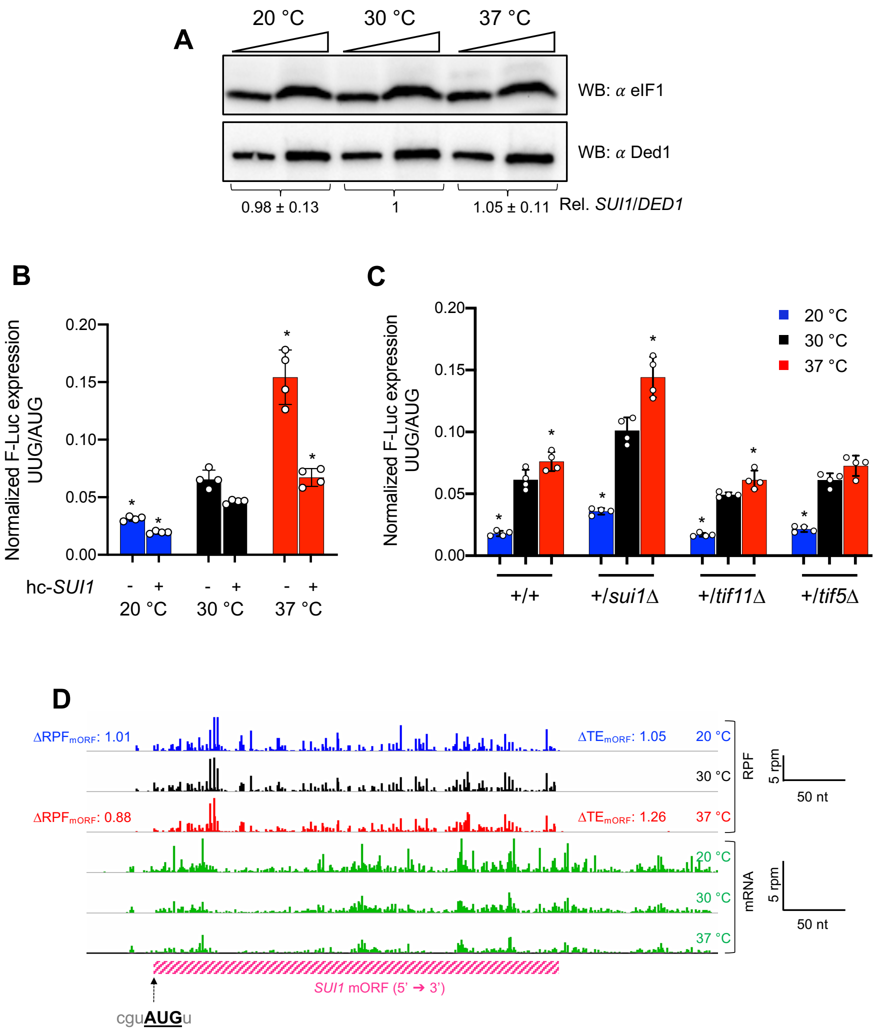

(A) BY4741 cells harboring F-Luc reporter (UUG) were cultured at either 20 °C, 30 °C or 37 °C to an A_{600} of 0.6 to 0.8 and whole cell extracts were subjected to Western blot analysis using antibodies against eIF1 and Ded1 (loading control). Extracts were loaded in two amounts differing by a factor of two. eIF1 western blot signal was normalized to that of Ded1, to calculate relative *SUI1* levels, which was then set as 1 for 30 °C and the relative *SUI1* expression at 20 °C or 37 °C was calculated. The values represent the average (±SD) from 4 independent experiments. **(B)** F-Luc expression was measured in a strain H3984 (hc-*SUI1*) and compared with wild-type (BY4741). (**C**) F-Luc expression was measured at 20 °C, 30 °C or 37 °C in haplo-insufficient diploids for eIFs 1 (+/*sui1Δ*), 1A (+/*tif11Δ*), and 5 (+/*tif5Δ*), and compared with diploid wild-type

BY4743 (+/+).For B and C: the values represent the average (±SD) from 4 independent experiments. The asterisk denotes p-values < 0.05 calculated by Student's *t*-test when compared with 30 °C. **(D)** Ribosome-protected fragments (RPFs) and mRNA reads (RNA-seq) on the *SUI1* gene in cells cultured at either 20, 30 or 37 °C, in units of rpm (reads per million mapped reads). The RPF reads are shown in blue (20 °C), black (30 °C) or red (37 °C). The mRNA reads at each temperature are shown in green. The dashed arrow represents the start site of the mORF of the *SUI1* gene along with its context nucleotides. The changes observed in RPF counts and TE for the mORF of eIF1 are shown in blue (20 °C) and red (37 °C).

Figure S3) Ribosome profiling at multiple growth temperatures.

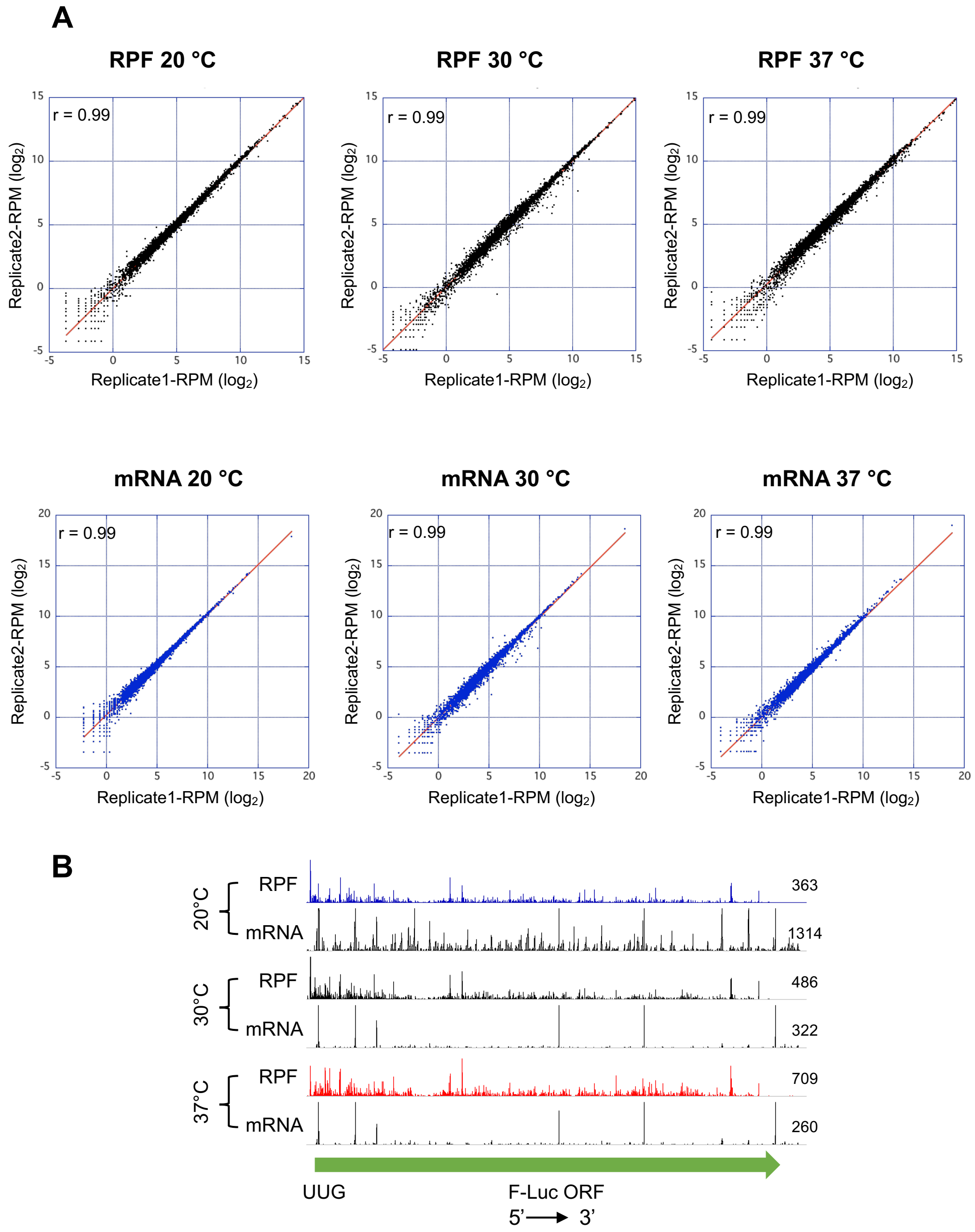

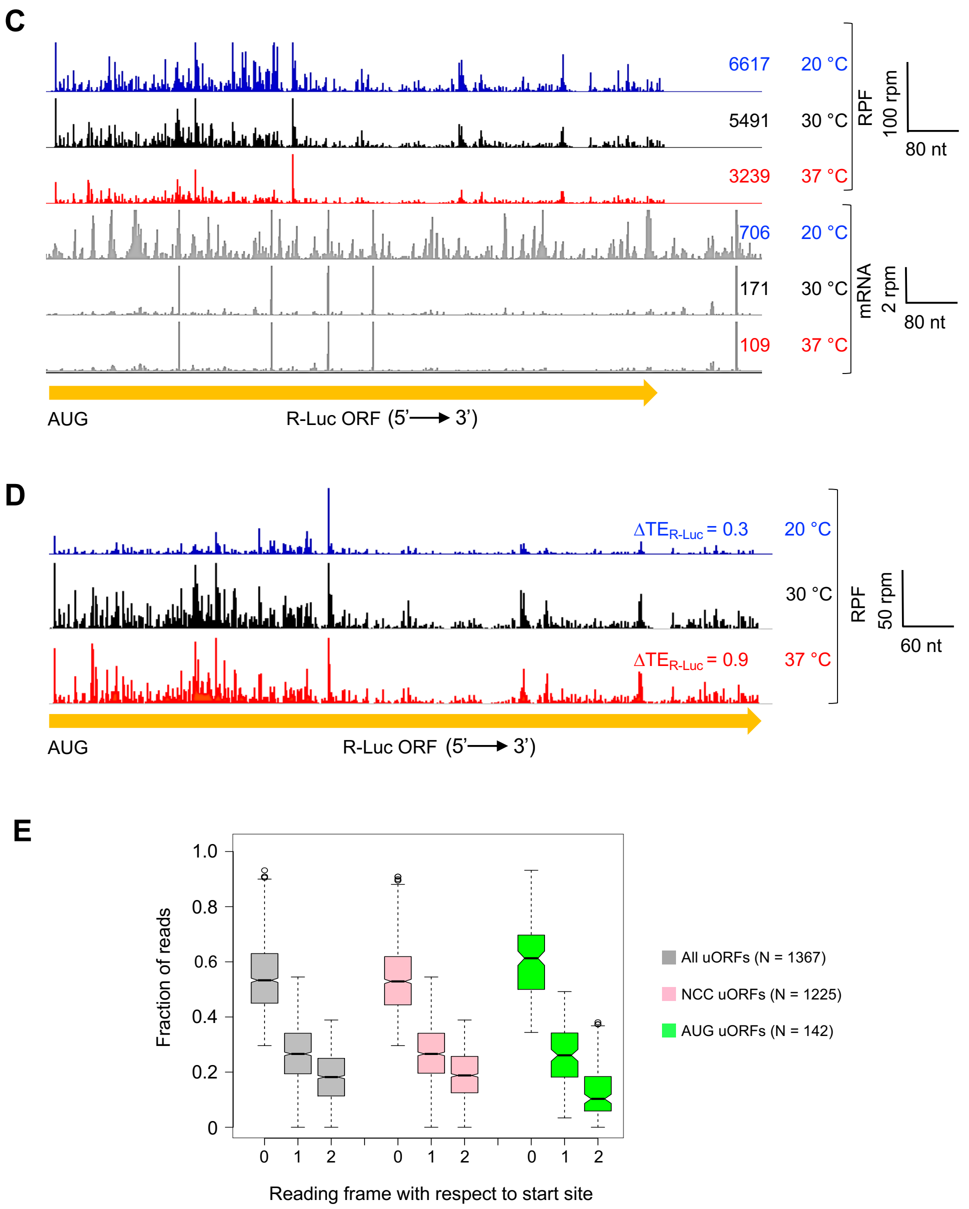

(A) Data reproducibility of ribosomal footprint and mRNA densities at multiple temperatures. Scatterplots of ribosomal densities [ribosome protected fragments (RPF) reads] in top panels and mRNA densities (RNA-seq reads) in bottom panels at 20, 30 and 37 °C. The densities were represented by the reads mapped to the coding region of a gene per million mapped reads in the individual library of a biological replicate. The Pearson correlation coefficients (r values) calculated for all genes (n~5400) are shown. **(B, C)** RPF and mRNA reads on the F-Luc^{UUG} reporter **(B)** and R-Luc^{AUG} reporter **(C).** Reads were normalized to the total number of mapped reads at each temperature. RPF reads for 20, 30 and 37 °C are shown in blue, black and red, respectively, while the mRNA reads are shown in gray for all the temperatures. The numbers at the end of each

wiggle track on the right indicate the average read densities (reads per million, RPM) of the two replicates. For the RNA density calculations, the presumed PCR jackpot peaks from the mRNA traces were removed. The numbers on each wiggle track indicate the average read densities (reads per million, RPM) of the two replicates. **(D)** Ribosome-protected fragments (RPFs) on the R-Luc^{AUG} reporter mRNA in cells cultured at 20, 30 or 37 °C, in units of rpm (reads per million mapped reads from two replicates at each temperature). The RPF-tracks were normalized to the mRNA levels (see methods) at each temperature to reflect the changes in translation efficiencies ($\Delta TE_{R\text{-}Luc}$). **(E)** Boxplot analysis of the distribution of RPF reads in each of the three reading frames of all the uORFs identified in this study. The fraction of reads in each frame of each uORF is plotted.

Figure S4) Changes in growth temperature lead to changes in translation of uORFs.

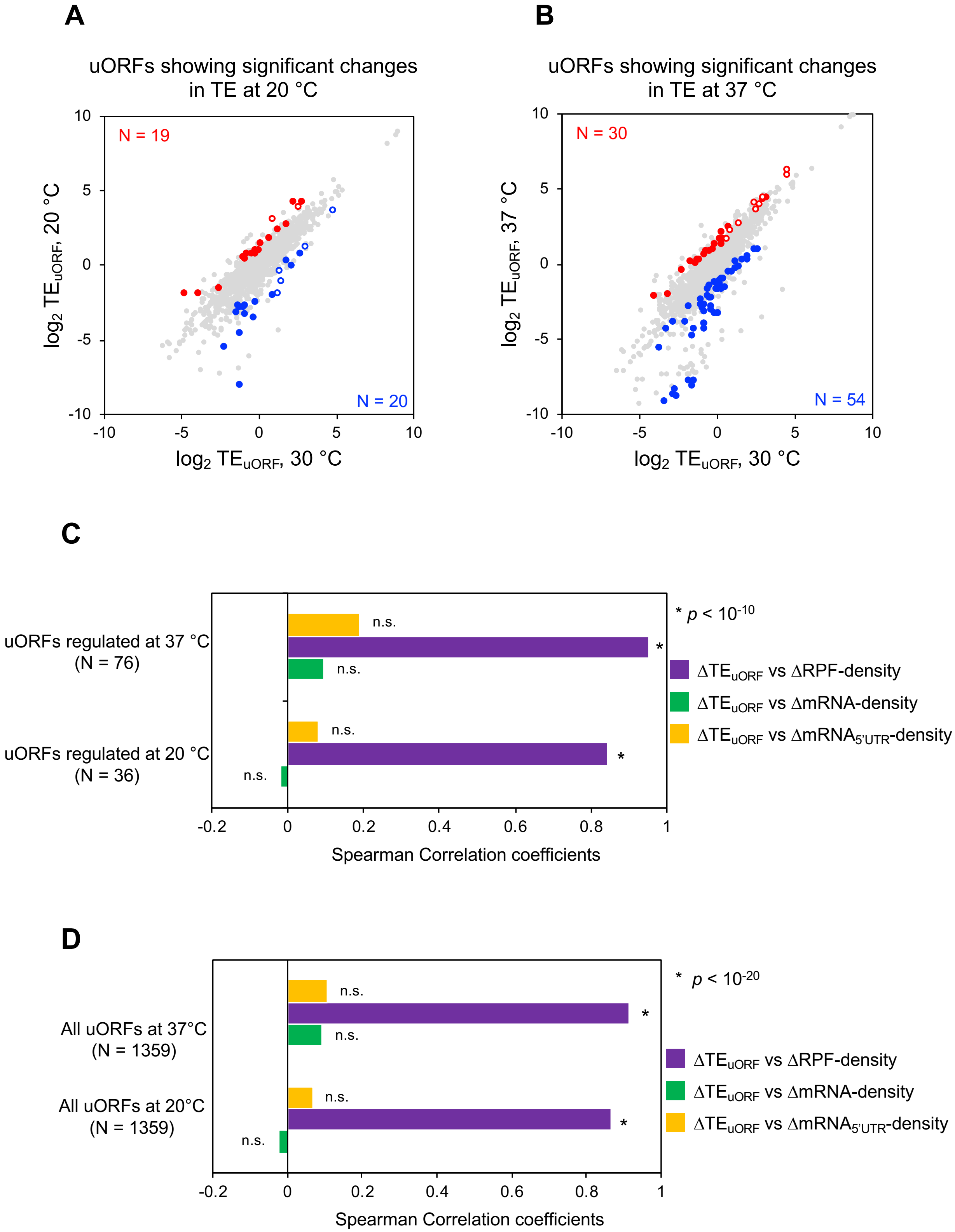

E

20 °C	ΔTE vs ΔRPF-density	ΔTE vs ΔmRNA-density	ΔTE vs ΔmRNA$_{5'UTR}$-density
All AUG uORFs	0.83 *	-0.26	-0.04
All NCC ORFs	0.87 *	0.01	0.07

* $p < 10^{-30}$

F

37 °C	ΔTE vs ΔRPF-density	ΔTE vs ΔmRNA-density	ΔTE vs ΔmRNA$_{5'UTR}$-density
All AUG uORFs	0.86 *	-0.07	0.2
All NCC uORFs	0.91 *	0.05	0.08

* $p < 10^{-30}$

(A) Scatterplot of TE_{uORF} for cells grown at 30 and 20 °C showing uORFs exhibiting ≥ 2-fold changes (repression or activation) in TE_{uORF} and at a False Discovery Rate (FDR) < 0.1. Note that these cut-offs are different than those in Figure 4A, B, which show TEs of uORFs exhibiting ≥ 2-fold changes in both TE_{uORF} *and* relative TE (TE_{uORF}/TE_{mORF}) at a False Discovery Rate (FDR) < 0.1. Red and blue circles represent such uORFs whose translation is activated or repressed respectively. The open circles are AUG uORFs while the filled circles are NCC uORFs. This plot differs from the plots in Figure 4 A and B (iii) in that no filter was applied here to ensure that the TE of the uORF changes more than the TE of the mORF. **(B)** Same as in **A**, except; scatterplot of TE_{uORF} for cells grown at 30 and 37 °C. **(C-F)** Spearman correlation coefficient analysis between ΔTE_{uORF} versus Δribo-density$_{uORF}$, ΔTE_{uORF} versus ΔmRNA-density$_{mORF}$ and ΔTE_{uORF} versus ΔmRNA-density$_{5'-UTR}$ for the all translated uORFs and regulated uORFs. Cases in which the two-sided p-value is less than 10^{-10} **(C)**, 10^{-20} **(D)** or 10^{-30} **(E, F)** are denoted with an asterisk. A high Spearman's coefficient value indicates a strong and directional monotonic relationship between the two variables tested. **(C)** uORFs showing temperature-dependent regulation of translation [as in Figures 4A and 4B, highlighted in red or blue in panel (iii)] are analyzed here. Regulated uORFs (activated or repressed) at 20 °C (N = 36) or at 37 °C (N = 76) were used to calculate the Spearman's correlation coefficients. **(D)** Same as in **C**; except with all translated uORFs (N = 1359). **(E, F)** Same as in **(D)** except all translated uORFs were analyzed [AUG uORFs (N = 136) and NCC uORFs (N = 1223)].

Figure S5) Analysis of translation efficiency changes of conserved uORFs upon changes in growth temperature.

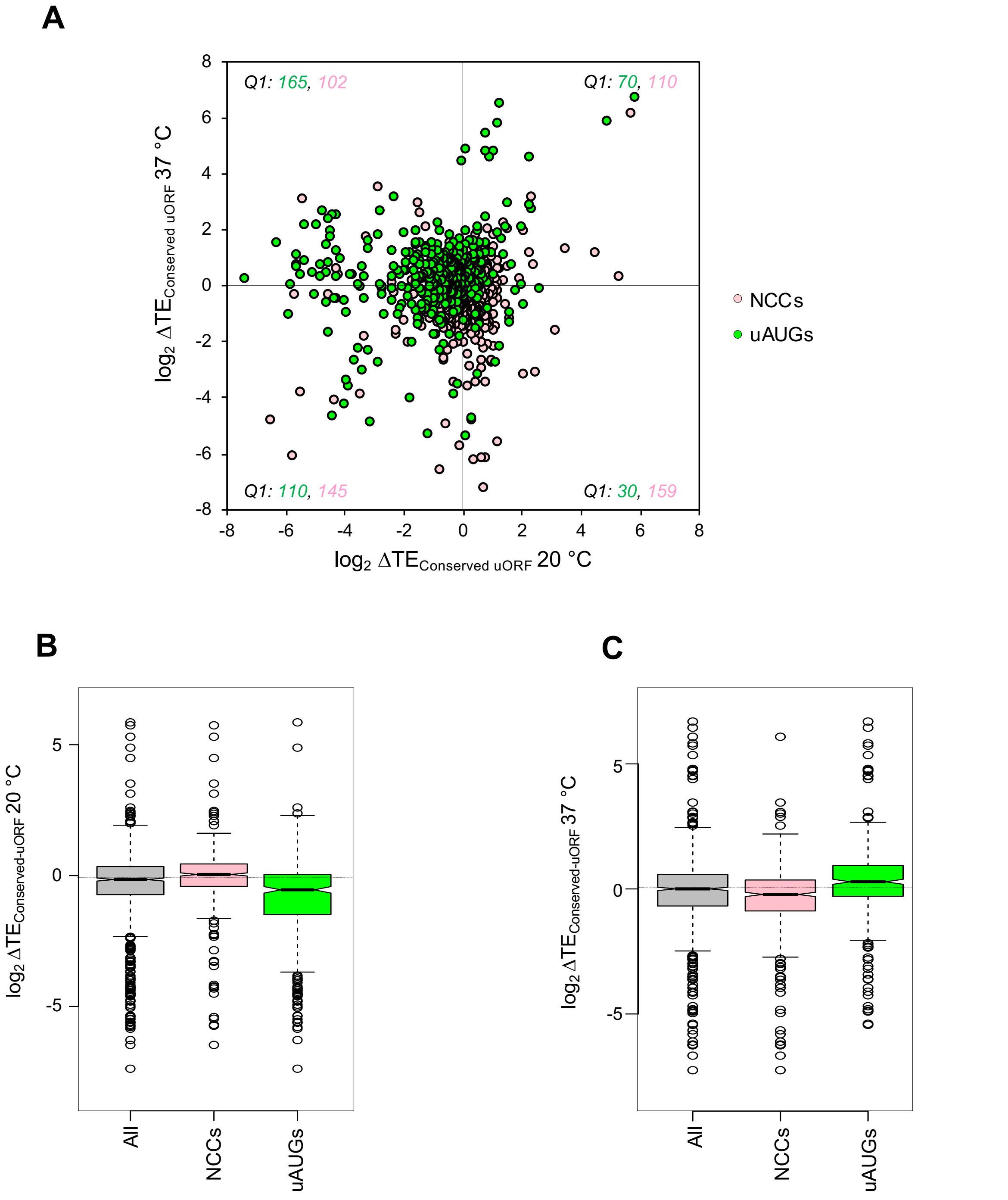

A) Translated uORFs shown to be conserved previously (see Results section) were used here for scatterplot analysis between changes in TEs at 20 °C ($\Delta TE_{Conserved\text{-}uORF}$ 20 °C) and 37 °C

($\Delta TE_{Conserved\text{-}uORF}$ 37 °C). The plot is divided into four quadrants (Q1 to Q4) based on the directionality of TE changes. The numbers in each quadrant represent the number of uORFs present in that quadrant; shown in pink for NCC uORFs and in green for AUG uORFs. **(B, C)** Boxplot analysis of $\Delta TE_{Conserved\text{-}uORF}$ for the conserved uORFs analyzed in Additional file 1: Figure S5A). *All* : 891 conserved uORFs whose ΔTE values could be calculated using the dataset generated in this study; *NCCs*: 516 conserved NCC uORFs; *uAUGs*: 375 conserved AUG uORFs. The dotted horizontal line represents the median ΔTE for '*All*'. **(B)** Analysis done for TE changes at 20 °C with respect to 30 °C. **(C)** Analysis done for TE changes at 37 °C with respect to 30 °C.

Figure S6) Analysis of the properties of various sets of uORFs and the mRNAs harboring them.

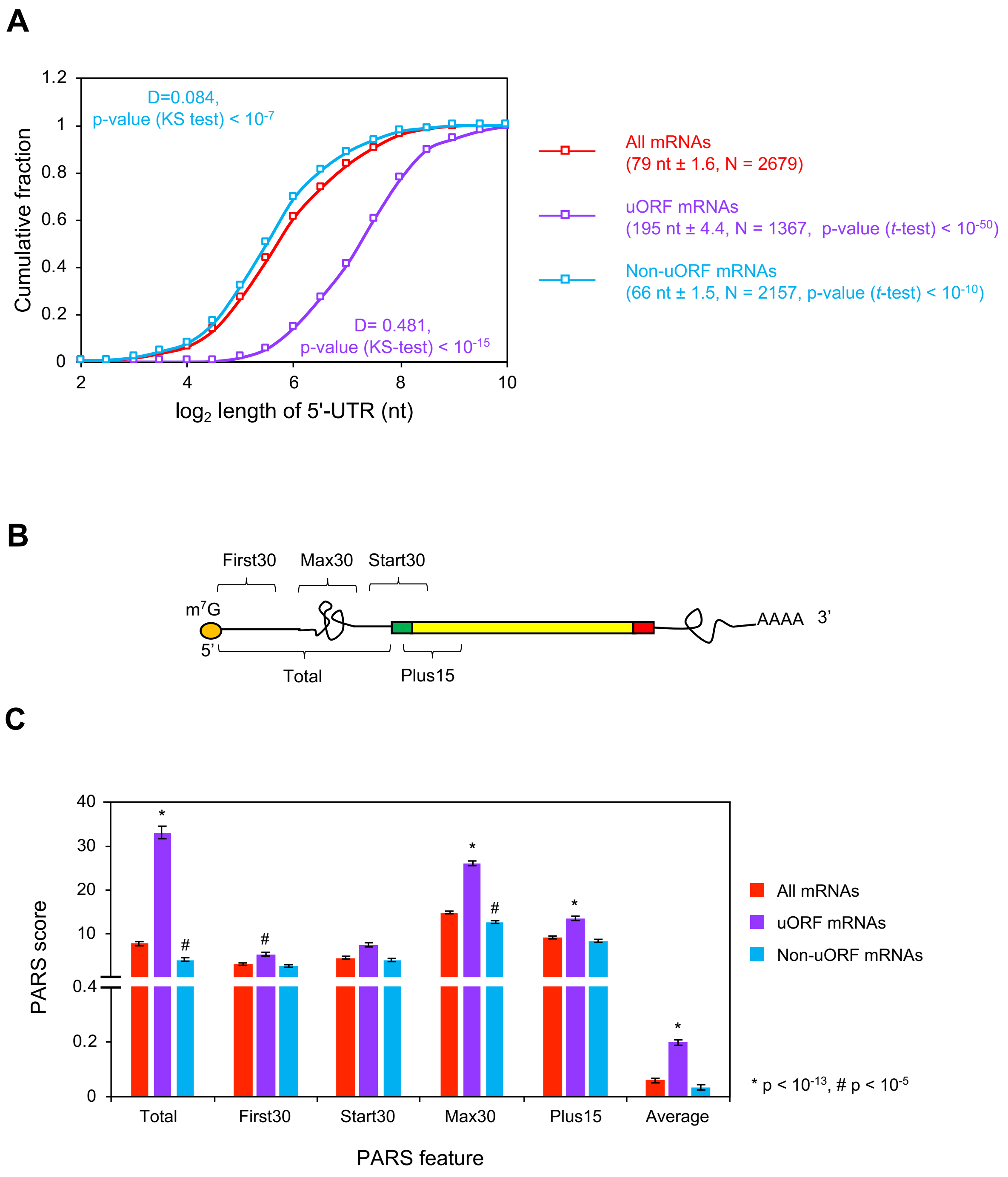

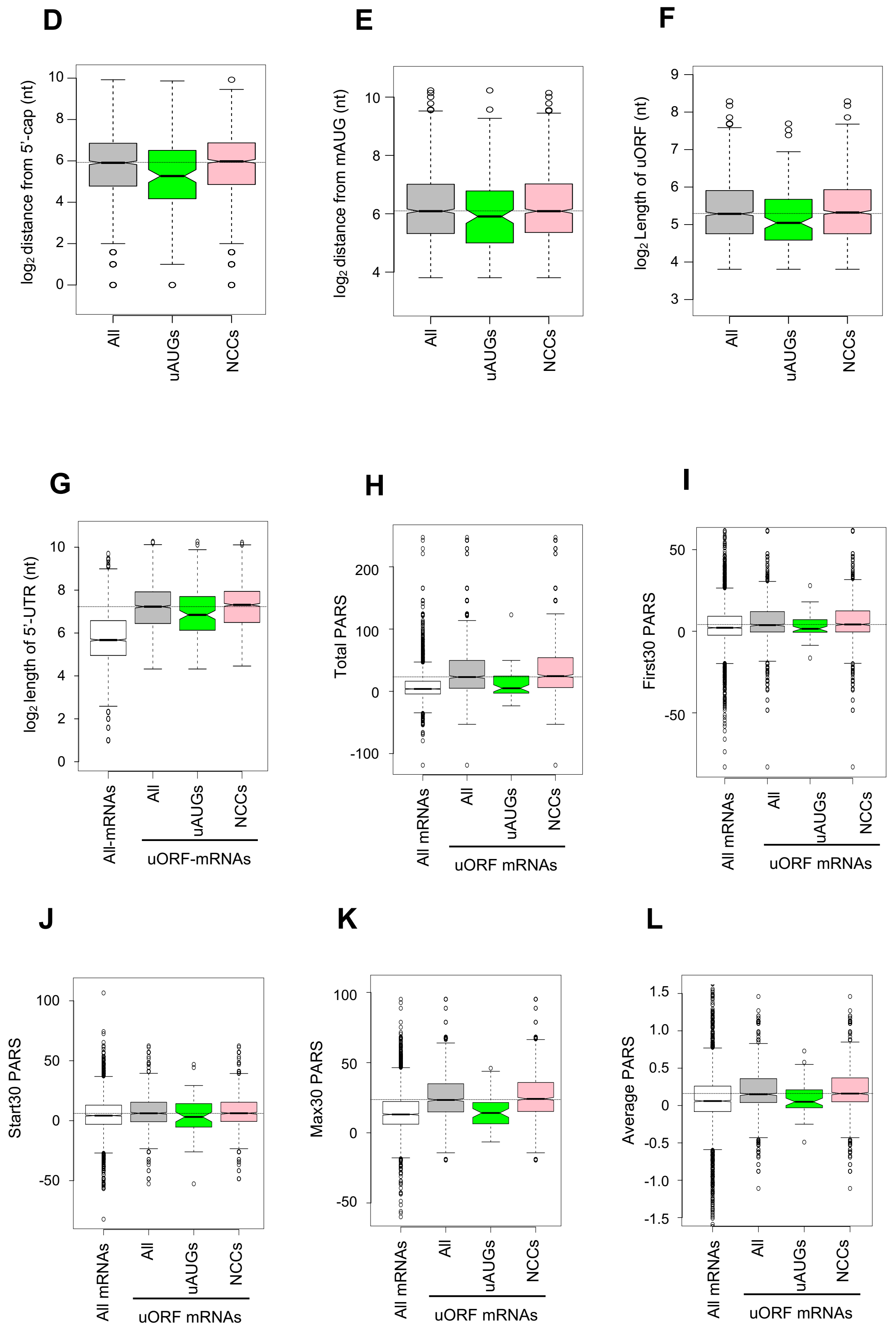
D
log2 distance from 5'-cap (nt)
All
uAUGs
NCCs
E
log2 distance from mAUG (nt)
All
uAUGs
NCCs
F
log2 Length of uORF (nt)
All
uAUGs
NCCs
G
log2 length of 5'-UTR (nt)
All-mRNAs
All
uAUGs
NCCs
uORF-mRNAs
H
Total PARS
All mRNAs
All
uAUGs
NCCs
uORF mRNAs
I
First30 PARS
All mRNAs
All
uAUGs
NCCs
uORF mRNAs
J
Start30 PARS
All mRNAs
All
uAUGs
NCCs
uORF mRNAs
K
Max30 PARS
All mRNAs
All
uAUGs
NCCs
uORF mRNAs
L
Average PARS
All mRNAs
All
uAUGs
NCCs
uORF mRNAs

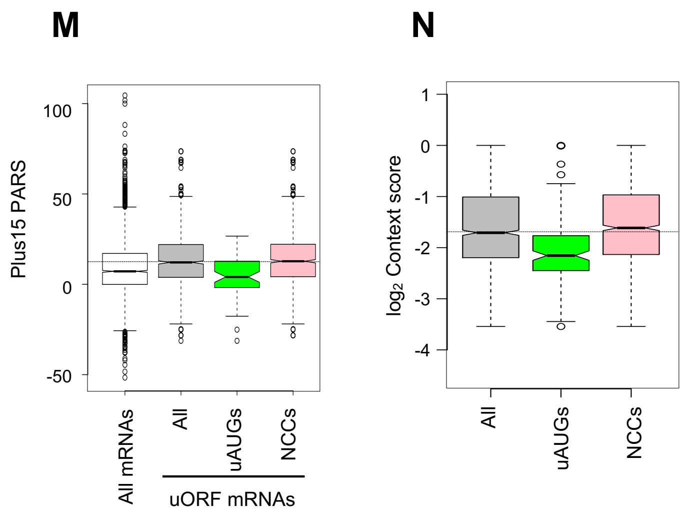

(A-C) 5'-UTRs of mRNAs with translated uORFs are longer and more structured than the genomic average. **(A)** Cumulative distribution of 5'-UTR lengths for all mRNAs (red), mRNAs with evidence of at least one translated uORF (purple), or mRNAs with no evidence of translated uORFs (blue). The average 5'-UTR length (in nt) ± SEM, number of mRNAs in each set and p-value by Student's *t*-test when compared with *All mRNAs* are shown in parentheses. The maximum difference in cumulative fraction (D) and p-value by Kolmogorov-Smirnov test (when compared with *All mRNAs*) are also shown. **(B)** mRNA intervals used for calculating PARS scores. **(C)** Mean PARS scores (± SEM) for each segment for *All mRNAs* (green, N~2700), *uORF-mRNAs* (purple, N~1020), *non-uORF mRNAs* (pink, N~2160) (if PARS scores were available). Student's t-test p values are shown compared to *All mRNAs*. **(D-F)** Boxplot analyses of intrinsic properties of mRNAs with *All* translated uORFs [described in Figure 3A (N = 1367)], *uAUG* uORFs (N = 142), and *NCC* uORFs (N = 1225). (**D)** *Distance from 5'-cap* to the uORF start codon. **(E)** *Distance from mAUG* to the uORF start codons. **(F)** *Length of uORF*. **G)** 5'-UTR lengths for *All mRNAs* (N = 2679); *All uORF mRNAs* (N = 1367); *AUG uORF mRNAs* (N = 142); and *NCC uORF mRNAs* (N = 1225). **(H-M)** PARS secondary structure formation propensities for the same sets of mRNAs as in (D): *All mRNAs* (n = 2679); *All uORF mRNAs* (N = 1024); *AUG uORF mRNAs* (N = 59); *NCC uORF mRNAs* (N = 965). **(N)** uORF start codon context scores for the same sets of mRNAs as in (D). The dotted horizontal line in each boxplot represents the median value of *All uORFs*.

Figure S7) Analysis of uORFs showing highest changes in translation at 20 °C and 37 °C.

A

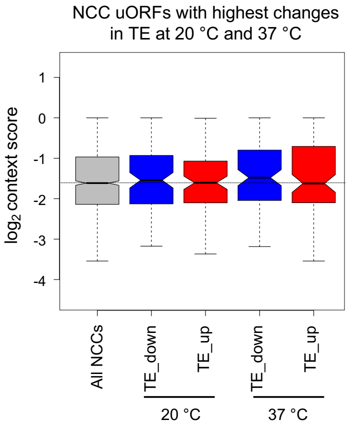

B

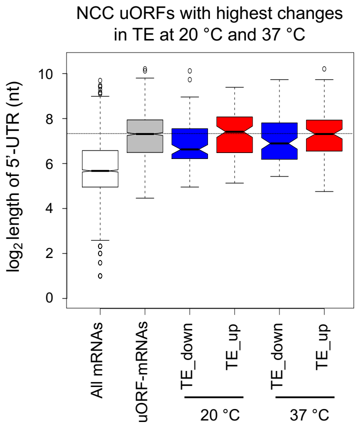

C

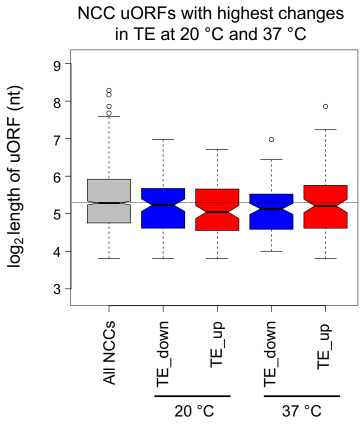

A) Boxplot analysis of uORF start codon context scores for the sets of regulated uORFs described in Figure 7A. *All NCCs* represent all translated NCC uORFs whose context scores were available (N = 1206). The dotted horizontal line represents the median context score for *All NCCs*. For *TE_down* or *TE_up* (20 °C or 37 °C) sets, at least 98/100 uORFs' context scores were available. **(A)** Boxplot analysis of the lengths of 5'-UTRs of the mRNAs corresponding to various sets of uORFs described in Figure 7A. *All* represents ~2700 mRNAs whose 5'-UTR length information is available from PARS dataset. *uORF-mRNAs* represents mRNAs with NCC uORFs (N = 1223) whose ΔTE_{uORF} could be determined and for which 5'-UTR length information was available. The dotted horizontal line represents the median length of the 5'-UTRs of *uORF-mRNAs*. **(B)** Boxplot analysis of the lengths of uORFs (in nucleotides) in different datasets described in Figure 7A. *All*

represents all the uORFs identified in this study starting with NCC and whose ΔTE_{uORF} could be measured (N = 1223). The dotted horizontal line represents the median length of *All NCCs*.

Figure S8) Boxplot analyses of 5’-UTR features for the most highly regulated NCC uORFs.

NCC uORFs with highest changes in TE at 20 °C and 37 °C

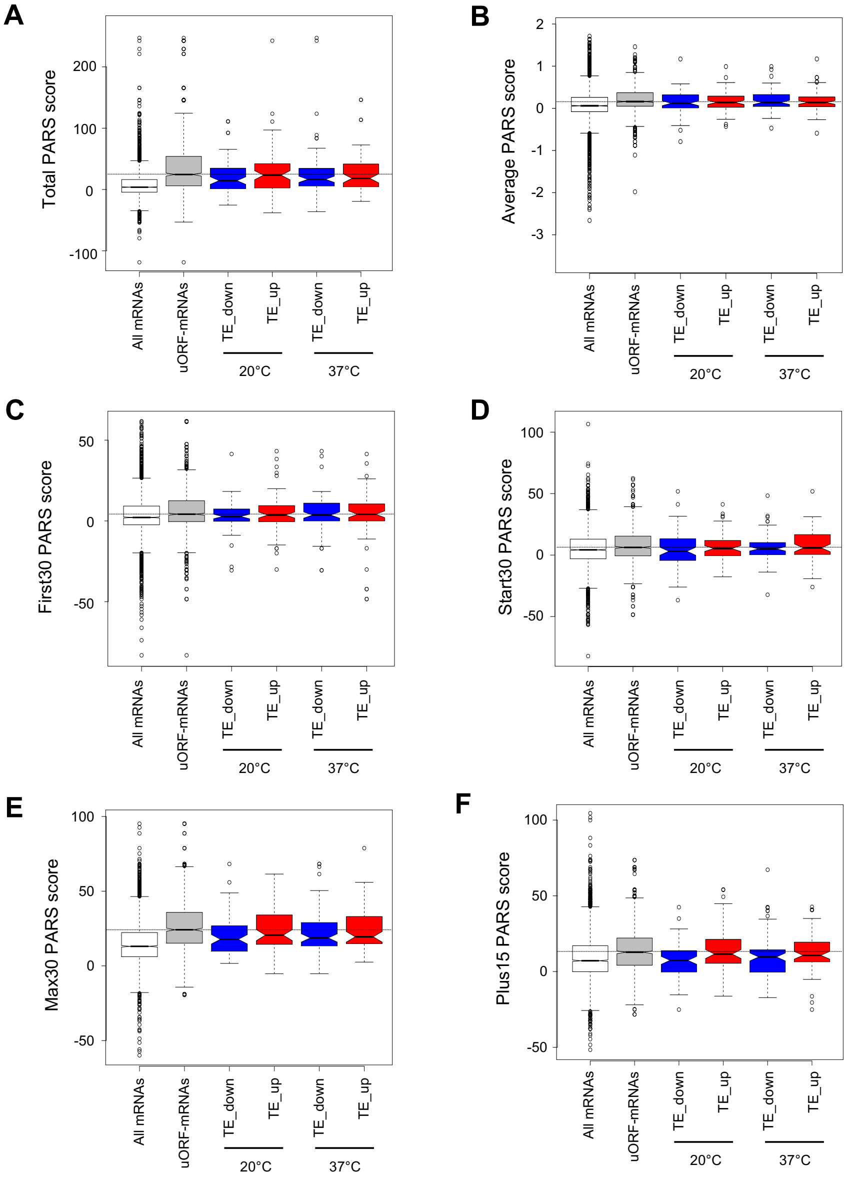

(A-F) Boxplot analysis of PARS scores of the 5’-UTRs of TE_up and TE_down NCC uORF mRNAs (described in Figure 7A). *All mRNAs* represent data from 2679 mRNAs whose PARS scores are available. *uORF-mRNAs* represent mRNAs with evidence of at least one translated NCC uORF identified using the pipeline described in Figure 3A (N = 1223) and whose PARS data are available (N = 965/1223). *TE_down* and *TE_up* (at either 20 °C or 37 °C) represent the most highly regulated uORFs as described in Figure 7A. PARS data were available for at least 65/100 uORFs in each category. The dotted horizontal line represents the median PARS score for each

feature calculated for the 5'-UTRs of *uORF-mRNAs*. **(A)** Analysis for total PARS score. **(B)** Analysis for average PARS score. **(C)** Analysis for First30 PARS score. **(D)** Analysis for Start30 PARS score. **(E)** Analysis for Max30 PARS score. **(F)** Analysis for Plus15 PARS score. PARS features are as defined in Additional file 1: Figure S6B.

Figure S9) Analysis of TE-changes of mORFs.

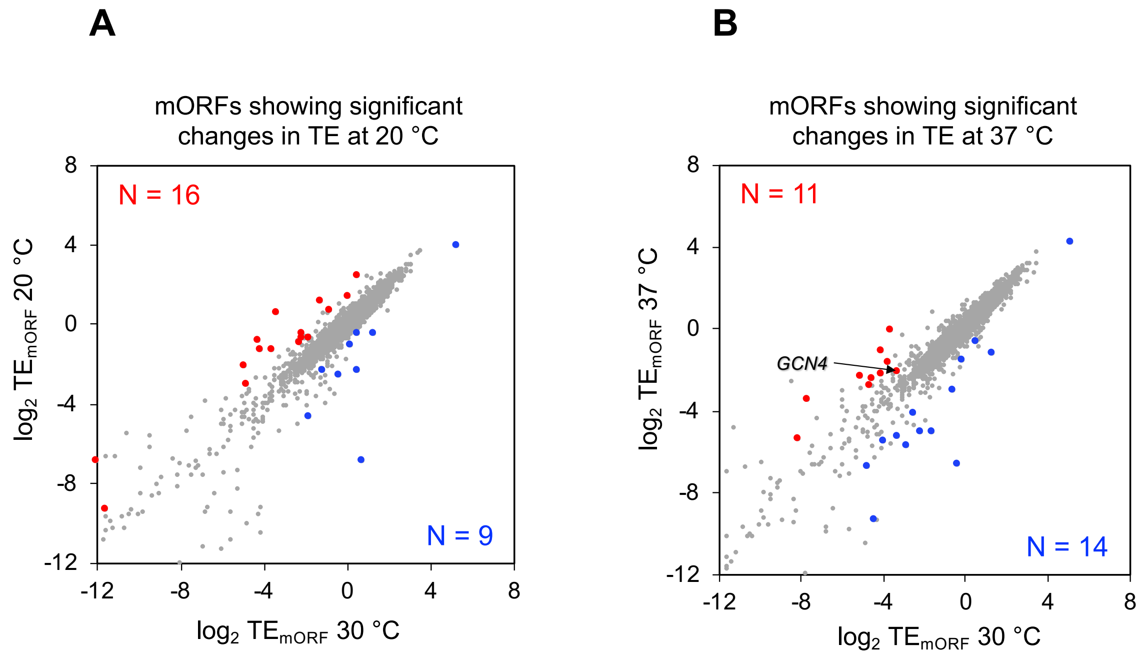

(A) Scatterplot of translational efficiencies (TEs) of mORFs in cells cultured at 30 °C and 20 °C. mORFs exhibiting ≥ 2-fold changes in TE in cells cultured at 20 °C and at FDR < 0.1 are shown in blue circles (repressed) or in red circles (activated). **(B)** Same as in **A**, except translational efficiencies (TEs) of mORFs of cells cultured at 30 °C and 37 °C is plotted.

Figure S10) Wiggle track images of examples of mRNAs that show temperature-dependent changes in translation of one or more uORF and the mORF.

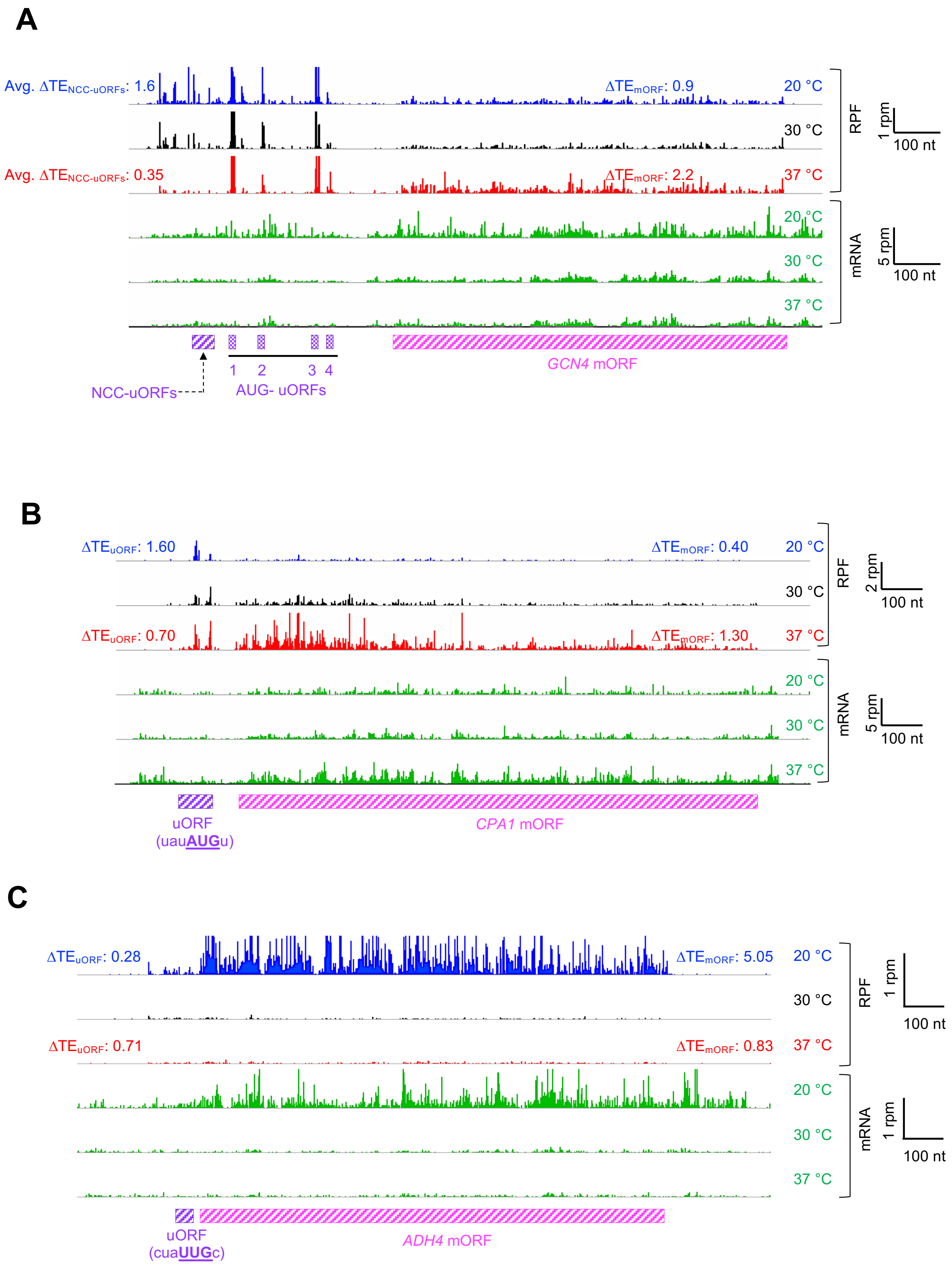

D

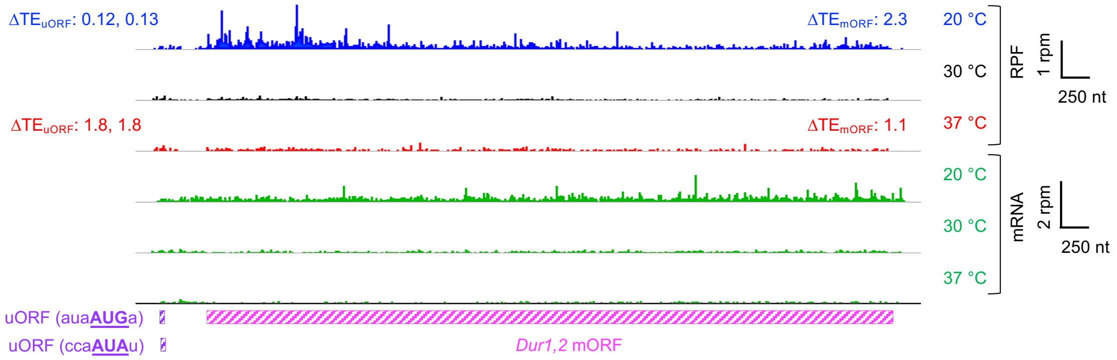
ΔTE_{uORF}: 0.12, 0.13
ΔTE_{mORF}: 2.3
ΔTE_{uORF}: 1.8, 1.8
ΔTE_{mORF}: 1.1
20 °C
30 °C
37 °C
RPF
1 rpm
250 nt
20 °C
30 °C
37 °C
mRNA
2 rpm
250 nt
uORF (auaAUGa)
uORF (ccaAUAu)
Dur1,2 mORF

E

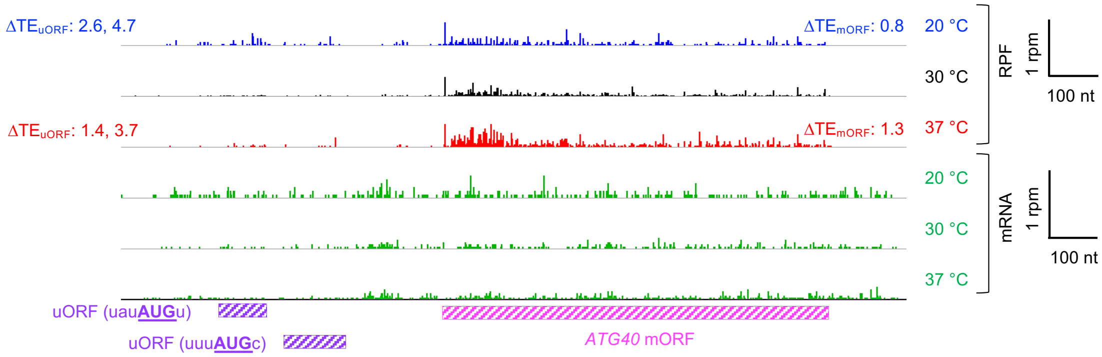
ΔTE_{uORF}: 2.6, 4.7
ΔTE_{mORF}: 0.8
ΔTE_{uORF}: 1.4, 3.7
ΔTE_{mORF}: 1.3
20 °C
30 °C
37 °C
RPF
1 rpm
100 nt
20 °C
30 °C
37 °C
mRNA
1 rpm
100 nt
uORF (uauAUGu)
uORF (uuuAUGc)
ATG40 mORF

F

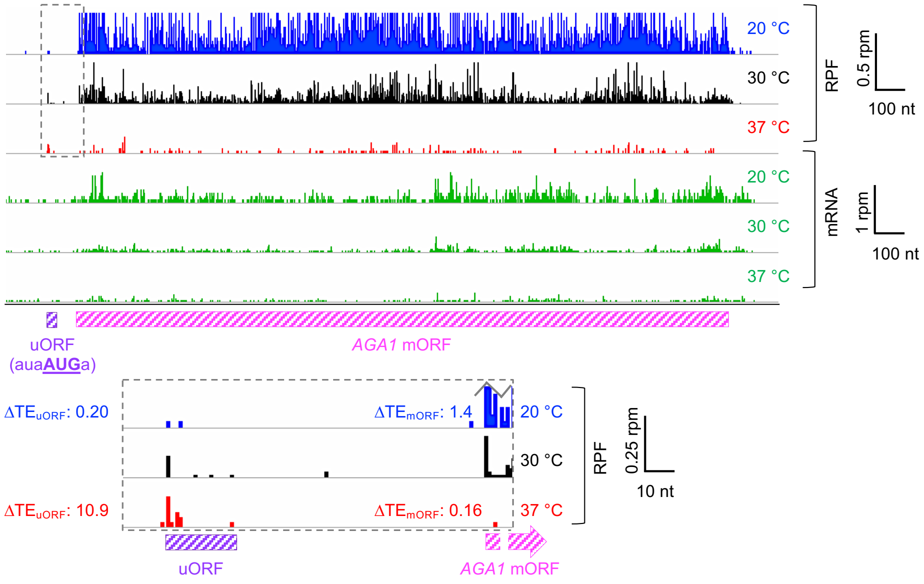
20 °C
30 °C
37 °C
RPF
0.5 rpm
100 nt
20 °C
30 °C
37 °C
mRNA
1 rpm
100 nt
uORF
(auaAUGa)
AGA1 mORF
ΔTE_{uORF}: 0.20
ΔTE_{mORF}: 1.4
ΔTE_{uORF}: 10.9
ΔTE_{mORF}: 0.16
20 °C
30 °C
37 °C
RPF
0.25 rpm
10 nt
uORF
AGA1 mORF

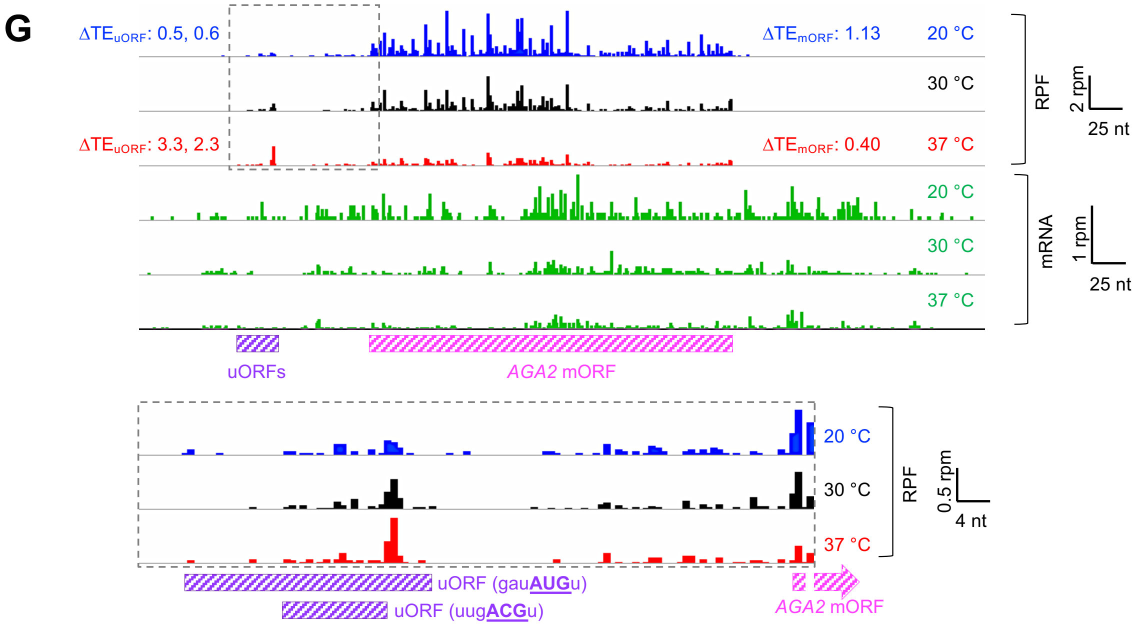
G
ΔTE uORF: 0.5, 0.6
ΔTE mORF: 1.13
20 °C
30 °C
ΔTE uORF: 3.3, 2.3
ΔTE mORF: 0.40
37 °C
RPF
2 rpm
25 nt
20 °C
30 °C
37 °C
mRNA
1 rpm
25 nt
uORFs
AGA2 mORF
20 °C
30 °C
37 °C
RPF
0.5 rpm
4 nt
uORF (gauAUGu)
uORF (uugACGu)
AGA2 mORF

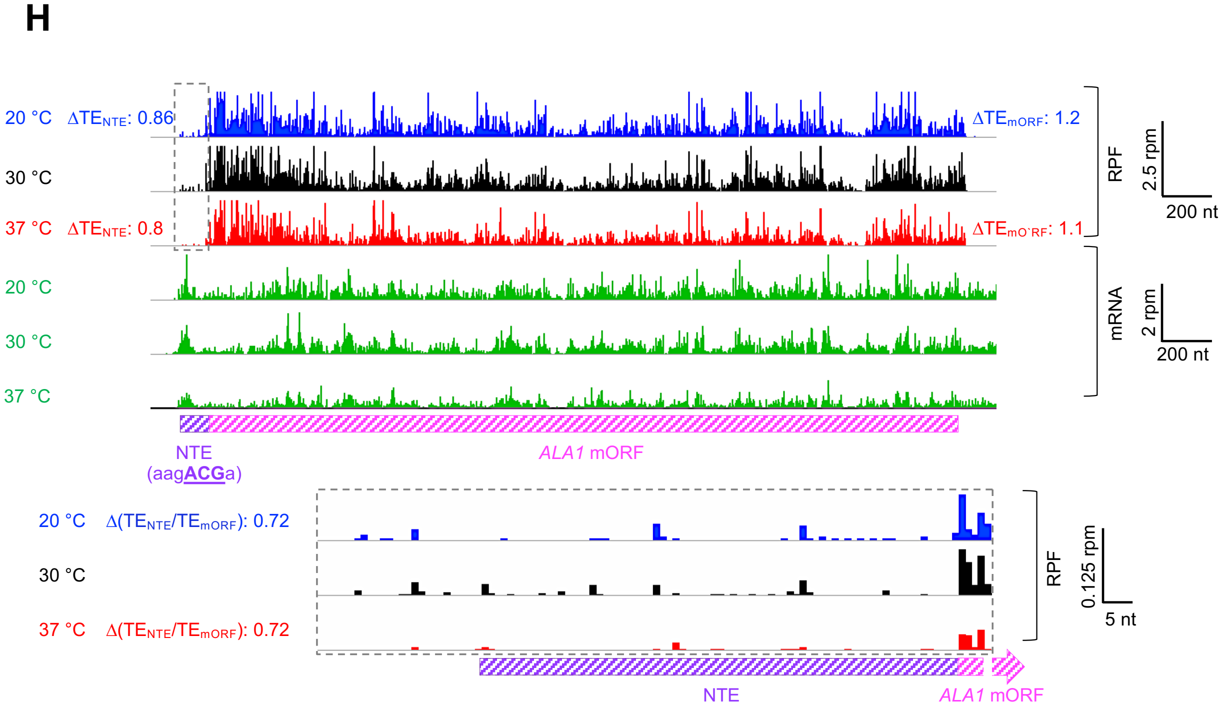
H
20 °C ΔTE NTE: 0.86
ΔTE mORF: 1.2
30 °C
37 °C ΔTE NTE: 0.8
ΔTE mO'RF: 1.1
RPF
2.5 rpm
200 nt
20 °C
30 °C
37 °C
mRNA
2 rpm
200 nt
NTE
(aagACGa)
ALA1 mORF
20 °C Δ(TE NTE/TE mORF): 0.72
30 °C
37 °C Δ(TE NTE/TE mORF): 0.72
RPF
0.125 rpm
5 nt
NTE
ALA1 mORF

I

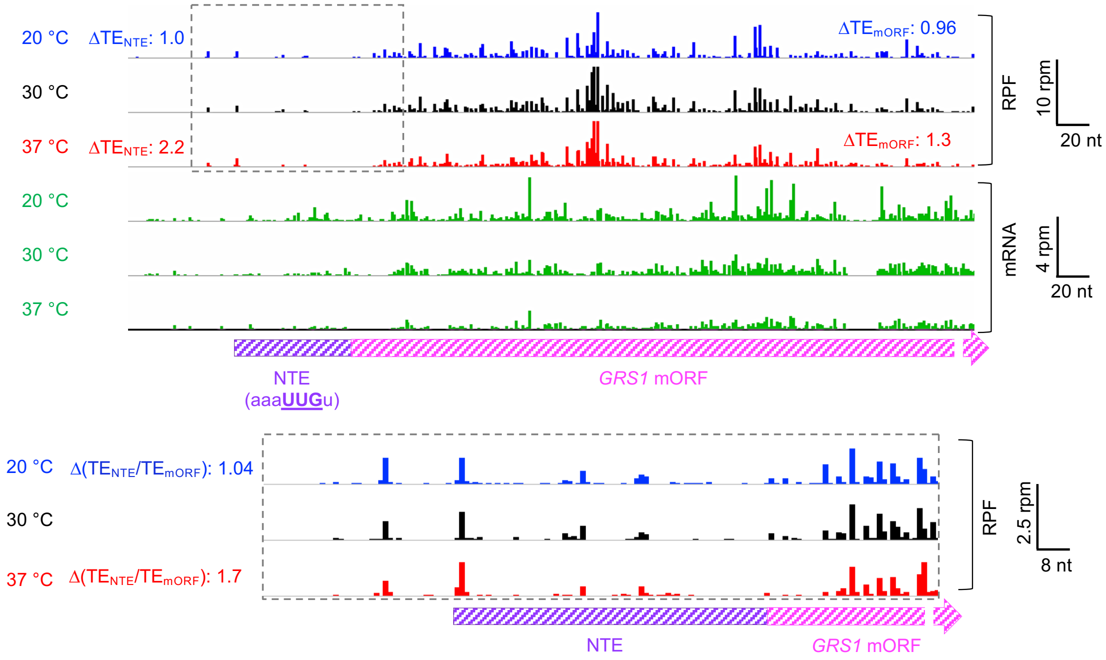

(A-I) Ribosome-protected fragments (RPFs) and mRNA reads are shown for data from cells cultured at either 20, 30 or 37 °C, in units of rpm (reads per million mapped reads) as in Figure 9A-I except the RPF-tracks were not normalized to the mRNA levels and the traces of mRNA reads from each temperature are shown in green.

Figure S11) uORF-mediated temperature-dependent regulation of *AGA1* mRNA.

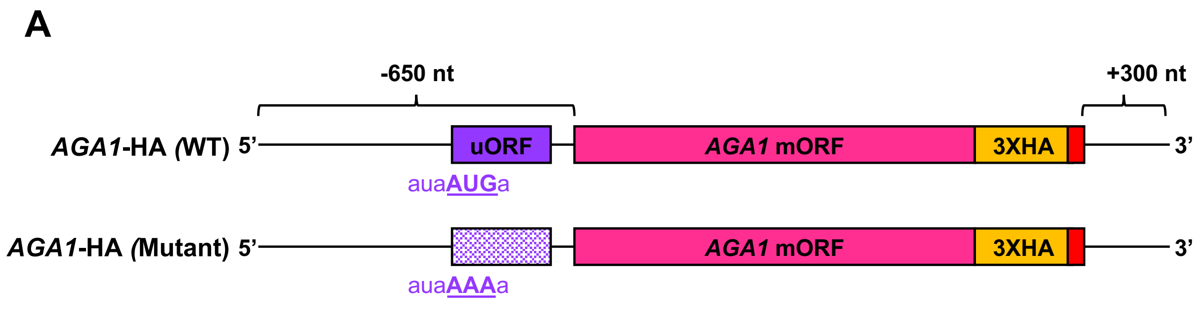

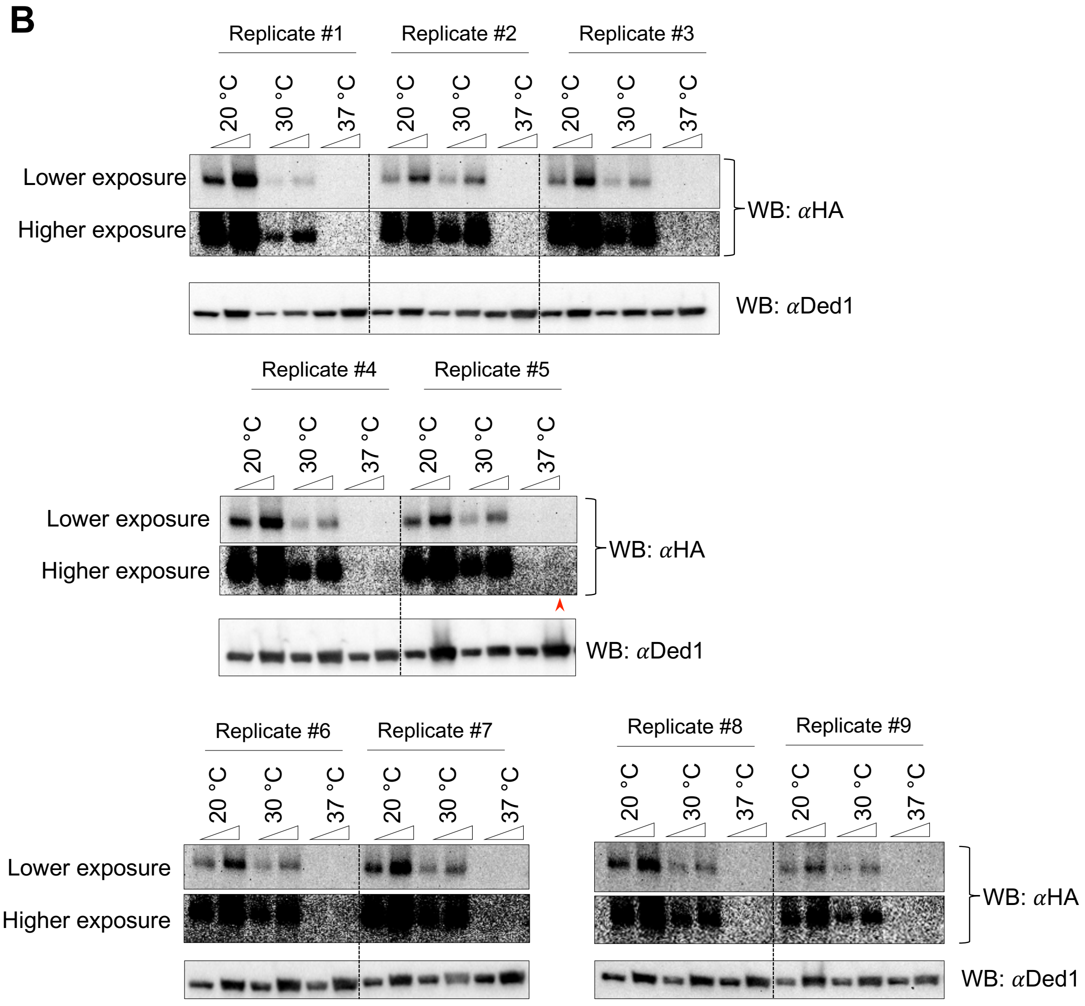

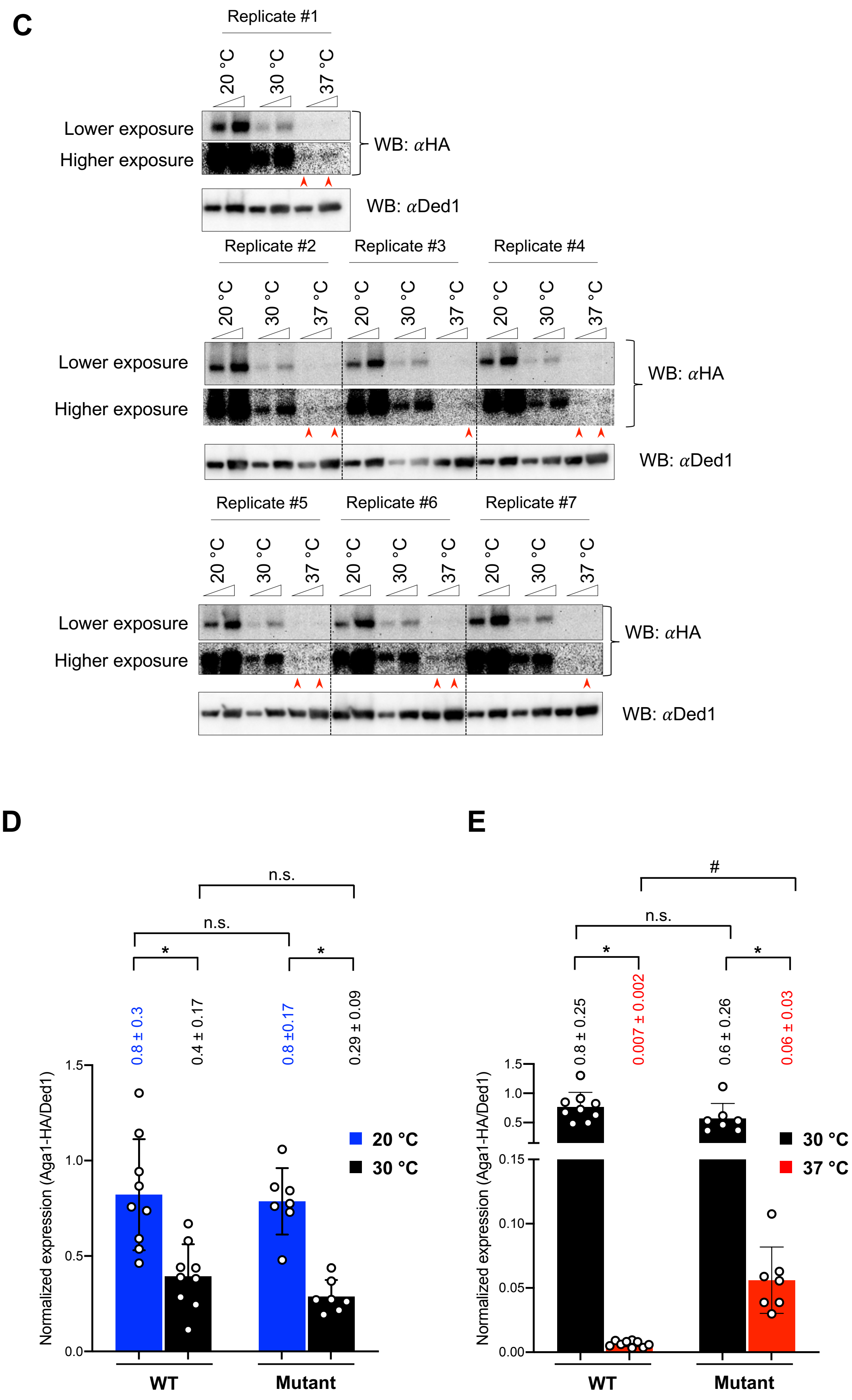
C
Replicate #1
20 °C
30 °C
37 °C
Lower exposure
Higher exposure
WB: αHA
WB: αDed1
Replicate #2
Replicate #3
Replicate #4
Lower exposure
Higher exposure
WB: αHA
WB: αDed1
Replicate #5
Replicate #6
Replicate #7
Lower exposure
Higher exposure
WB: αHA
WB: αDed1
D
n.s.
n.s.
*
*
0.8 ± 0.3
0.4 ± 0.17
0.8 ±0.17
0.29 ± 0.09
Normalized expression (Aga1-HA/Ded1)
1.5
1.0
0.5
0.0
20 °C
30 °C
WT
Mutant
E
#
n.s.
*
*
0.8 ± 0.25
0.007 ± 0.002
0.6 ± 0.26
0.06 ± 0.03
Normalized expression (Aga1-HA/Ded1)
1.5
1.0
0.5
0.15
0.10
0.05
0.00
30 °C
37 °C
WT
Mutant

(A) Schematics of the reporters used (see Methods and Additional file 1, Supplementary Table 2). Purple and pink boxes represent *AGA1* uORF and *AGA1* mORF, respectively. An orange box represents the 3XHA tag inserted just before the stop codon (shown in red box). **(B)** BY4741 cells harboring *AGA1*-HA (WT) plasmid, were cultured at either 20, 30, or 37 °C to an A_{600} of 0.6 to 0.8 and whole cell extracts were subjected to Western blot analysis using antibodies against HA and Ded1 (the loading control). Extracts were loaded in two amounts differing by a factor of two. For the HA Western blots, lower and higher exposures are shown to visualize any possible signal from the 37 °C cultures. The red arrowhead indicates the Aga1-HA band detected from lysates made from 37 °C cultures. The vertical black dotted lines separate the lysates made from each biological replicate. **(C)** Same as in **B**, except using cell extracts prepared from BY4741 cells harboring *AGA1*-HA (Mutant) plasmid, in which the uORF AUG codon was changed to an AAA codon. **(D, E)** Quantification of the western signal from **B** and **C**. The HA signal was normalized to that of Ded1 ("Normalized Expression"). The value over each column represents the average normalized expression (±SD) from multiple biological replicates (shown in open circles). **(D)** For both WT and mutant cases, the quantification of the HA signal for 20 and 30 °C lysates was done using the lower exposures. **(E)** Same as in **D,** except quantification of the HA signal for 30 and 37 °C lysates was done using the higher exposures for the HA signal. The '*' denotes p-value (*t*-test) < 0.005 when compared to 30 °C control. The '#' denotes p-value (*t*-test) < 0.005 when WT 37 °C and mutant 37 °C datasets were compared.
