# Supplementary material for: Temperature-dependent regulation of upstream open reading frame translation in S. cerevisiae
Source: BMC Biol. 2019 Dec 6;17:101. doi: 10.1186/s12915-019-0718-5 (PMC6898956; doi:10.1186/s12915-019-0718-5)
Supplement: Supplementary file 2 — Additional file 2: Table S1. Yeast strains used in this study. Table S2. Plasmids used in this study. Table S3. List of uORFs showing temperature-dependent translational regulation. Table S4. List of putative N-terminal extensions identified in this study. Table S5. List of datasets generated in this study and their GEO accession numbers. [file 12915_2019_718_MOESM2_ESM.pdf]

Additional file 2: Supplementary Tables 1 to 5

Supplementary Table 1. Yeast strains used in this study

Sr. No.	Strain	*Genotype*	Source/reference
1	BY4741	*MATa his3Δ1 leu2Δ0 met15Δ0 ura 3Δ0*	Open Biosystems
2	*upf1Δ*	*MATa his3 leu2 met15 ura3 upf1:KanMX6*	[see Methods]
3	BY4743	*MATa /a his3Δ/his3Δ1 leu2Δ0/leu2Δ0 LYS2/lys2Δ0 met15Δ0/MET15 ura3Δ0/ura3Δ0*	Open Biosystems
4	YNL244C (+/*sui1Δ*)	*MATa/a his3Δ/his3Δ1 leu2Δ0/leu2Δ0 LYS2/lys2Δ0 met15Δ0/MET15 ura3Δ0/ura3Δ0+/sui1Δ*	Open Biosystems
5	YMR260C (+/*tif11Δ*)	*MATa/a his3Δ/his3Δ1 leu2Δ0/leu2Δ0 LYS2/lys2Δ0 met15Δ0/MET15 ura3Δ0/ura3Δ0+/ tif11Δ*)	Open Biosystems
6	YPR041W (+/*tif5Δ*)	*MATa/a his3Δ/his3Δ1 leu2Δ0/leu2Δ0 LYS2/lys2Δ0 met15Δ0/MET15 ura3Δ0/ura3Δ0+/ tif5Δ*)	Open Biosystems

Supplementary Table 2. Plasmids used in this study

Sr. No.	Plasmid	Genotype
1	$pR^{AUG}FF^{AUG}$	Dual luciferase reporter R-Luc(AUG)-F-Luc(AUG) in *URA3* vector
2	$pR^{AUG}FF^{UUG}$	Dual luciferase reporter R-Luc(AUG)-F-Luc(UUG) in *URA3* vector
3	$pR^{AUG}FF^{CUG}$	Dual luciferase reporter R-Luc(AUG)-F-Luc(CUG) in *URA3* vector
4	$pR^{AUG}FF^{GUG}$	Dual luciferase reporter R-Luc(AUG)-F-Luc(GUG) in *URA3* vector
5	$pR^{AUG}FF^{ACG}$	Dual luciferase reporter R-Luc(AUG)-F-Luc(ACG) in *URA3* vector
6	$pR^{AUG}FF^{AUC}$	Dual luciferase reporter R-Luc(AUG)-F-Luc(AUC) in *URA3* vector
7	$pR^{AUG}FF^{AUU}$	Dual luciferase reporter R-Luc(AUG)-F-Luc(AUU) in *URA3* vector
8	$pR^{AUG}FF^{AUA}$	Dual luciferase reporter R-Luc(AUG)-F-Luc(AUA) in *URA3* vector
9	p367	sc *URA3* HIS4(AUG)-lacZ
10	p391	sc *URA3* HIS4(UUG)-lacZ
11	hc-*SUI1*	*SUI1* on a high-copy plasmid (*LEU2* vector)
12	*AGA1*-HA (WT)	*AGA1* gene on a high copy plasmid (pRS426) with an insertion of in-frame 3XHA tag before the stop codon.
13	*AGA1*-HA (Mutant)	Same as #12 except the uORF start site in mutated from ATG to AAA.

Supplementary Table 3. List of uORFs showing temperature-dependent translational regulation.

Sr. No.	Gene name	Standard name	Position from mAUG	Context	Start codon	ΔTE_{uORF} 20 °C	ΔTE_{uORF} 37 °C
1	YBR208C	DUR1,2	-393	AUAAUGA	AUG	0.12	1.79
2	YDL073W	AHK1	-50	CGUAUGC	AUG	0.32	1.65
3	YGL032C	AGA2	-108	GAUAUGU	AUG	0.47	3.29
4	YJL140W	RPB4	-92	N/A	AUG	0.18	1.51
5	YLR097C	HRT3	-31	CACAUGU	AUG	0.31	1.25
6	YBR015C	MNN2	-62	UCAAGGU	AGG	0.23	1
7	YBR208C	DUR1,2	-384	CCAAUAU	AUA	0.13	1.85
8	YER056C	FCY2	-31	AACAUCC	AUC	0.19	0.69
9	YDR505C	PSP1	-38	GCGGUGU	GUG	0.01	2.21
10	YBR283C	SSH1	-92	GGGUUGG	UUG	0.11	1.66
11	YCR053W	THR4	-32	UAUUUGU	UUG	0.28	0.72
12	YGL009C	LEU1	-54	AUAUUGA	UUG	0.3	0.76
13	YGL009C	LEU1	-48	AAAUUGA	UUG	0.36	0.81
14	YGL009C	LEU1	-38	UUUUUGU	UUG	0.32	0.84
15	YGL256W	ADH4	-61	CUAUUGC	UUG	0.28	0.71
16	YJL124C	LSM1	-93	GGAUUGC	UUG	0.25	1.28
17	YJL164C	TPK1	-118	UUUUUGU	UUG	0.37	1.27
18	YMR054W	STV1	-182	GUGUUGA	UUG	0.11	0.35
19	YNL287W	SEC21	-179	GAAUUGU	UUG	0.41	0.97
20	YPL023C	MET12	-33	GUGUUGG	UUG	0.11	1.02
21	YDL134C	PPH21	-158	UGAAUAG	AUA	2.18	1.4
22	YDL134C	PPH21	-134	AAACUGU	CUG	2.31	1.44
23	YDR072C	IPT1	-77	UAACUGU	CUG	2.65	2.7
24	YGR041W	BUD9	-139	AAUUUGA	UUG	2.46	1.38
25	YHR205W	SCH9	-339	UCGCUGG	CUG	2.95	0.88
26	YJL052W	TDH1	-60	GGUUUGA	UUG	2.07	0.82
27	YKL185W	ASH1	-85	CUUAUUA	AUU	2.28	1.08
28	YLR208W	SEC13	-29	AAGAUAA	AUA	2.68	0.28

29	YMR092C	AIP1	-31	UUAACGG	ACG	8.11	0.1
30	YOL040C	RPS15	-16	AAGAUAA	AUA	3.05	0.97
31	YOL109W	ZEO1	-19	ACUACGU	ACG	4	0.34
32	YOR136W	IDH2	-36	CAGAUCG	AUC	4.17	0.47
33	YPL184C	MRN1	-465	UCUCUGU	CUG	2.57	0.86
34	YPR036W-A	SPO24	-43	UUACUGA	CUG	2.79	0.31
35	YOR152C	ATG40	-452	UAUAUGU	AUG	2.58	1.37
36	YOR152C	ATG40	-323	UUUAUGC	AUG	4.67	3.68
37	YAL016W	TPD3	-50	CAACUGG	CUG	1.14	0.33
38	YAL040C	CLN3	-82	UUUCUGA	CUG	1.32	0.35
39	YAL040C	CLN3	-75	CCCAUAG	AUA	1.15	0.23
40	YBL047C	EDE1	-76	UAGAUUC	AUU	1.38	0.42
41	YBR067C	TIP1	-17	CCUCUGC	CUG	1.12	0.26
42	YBR082C	UBC4	-41	UCACUGA	CUG	1.39	0.36
43	YBR196C	PGI1	-20	AGAAUCA	AUC	0.58	0.49
44	YBR196C	PGI1	-38	GUCUUGC	UUG	0.63	0.48
45	YCR069W	CPR4	-50	AGAUUGG	UUG	1.05	0.3
46	YDR505C	PSP1	-94	GUAUUGA	UUG	0.62	0.01
47	YDR524C-B	YDR524C-B	-25	UCAAUCA	AUC	1.07	0.29
48	YEL009C	GCN4	-454	CUCAUCU	AUC	1.58	0.4
49	YEL009C	GCN4	-442	GCAAUCA	AUC	1.42	0.38
50	YEL009C	GCN4	-392	AGUAUCG	AUC	1.79	0.26
51	YER094C	PUP3	-32	AAAAUAU	AUA	0.74	0.19
52	YFL039C	ACT1	-80	AGGAUCU	AUC	0.9	0.49
53	YFL039C	ACT1	-23	AAGAUCG	AUC	1.26	0.46
54	YFR009W	GCN20	-46	AAAACGU	ACG	1.05	0.02
55	YGL077C	HNM1	-96	UGCAUAA	AUA	0.87	0.39
56	YGR054W	YGR054W	-54	UCGAUAA	AUA	1.36	0.11
57	YGR054W	YGR054W	-82	GUGUUGU	UUG	2.1	0.3
58	YGR189C	CRH1	-35	AAUAUAA	AUA	1.23	0.47
59	YGR240C	PFK1	-155	UAAAUAA	AUA	0.73	0.27
60	YGR281W	YOR1	-37	UUUAUAU	AUA	0.87	0.01
61	YHL034C	SBP1	-102	AAAAUAA	AUA	0.77	0.11
62	YHL034C	SBP1	-99	AUAAUCA	AUC	0.76	0.11

63	YKL096W	CWP1	-22	ACUACGA	ACG	2.52	0.11
64	YKL104C	GFA1	-64	UAUAUAG	AUA	1.18	0.01
65	YKL164C	PIR1	-17	CUAAUAG	AUA	1.46	0.36
66	YLL013C	PUF3	-133	ACAUUGA	UUG	0.49	0.02
67	YLL013C	PUF3	-172	CAUCUGG	CUG	0.93	0.15
68	YLR432W	IMD3	-83	UUUAUUC	AUU	1.17	0.27
69	YLR432W	IMD3	-40	AAUAUUA	AUU	1.42	0.3
70	YNL091W	NST1	-56	GCCAUUA	AUU	0.84	0.2
71	YNR001C	CIT1	-61	ACAAUUA	AUU	0.77	0.02
72	YNR051C	BRE5	-184	AAUCUGU	CUG	1.07	0.33
73	YNR055C	HOL1	-66	CUAACGA	ACG	0.58	0.1
74	YNR055C	HOL1	-60	AAUAUAA	AUA	0.59	0.1
75	YNR055C	HOL1	-359	ACGAUAA	AUA	0.88	0.25
76	YOL109W	ZEO1	-19	ACUACGU	ACG	4	0.34
77	YOR124C	UBP2	-50	AAAGUGG	GUG	1.23	0.09
78	YOR124C	UBP2	-44	GUAAUUA	AUU	1.26	0.13
79	YOR142W	LSC1	-164	ACACUGC	CUG	0.79	0.38
80	YOR142W	LSC1	-154	UAUAUAG	AUA	0.71	0.32
81	YOR298C-A	MBF1	-22	UUAACGA	ACG	0.6	0.24
82	YPL032C	SVL3	-65	AGUCUGA	CUG	0.53	0.32
83	YPL184C	MRN1	-649	GCAAUCA	AUC	0.7	0.33
84	YPL184C	MRN1	-343	UAUUUGA	UUG	0.83	0.38
85	YPR036W-A	SPO24	-43	UUACUGA	CUG	2.79	0.31
86	YPR124W	CTR1	-127	UAUUUGC	UUG	1	0.33
87	YPR181C	SEC23	-37	UAUCUGU	CUG	1.21	0.02
88	YPR191W	QCR2	-43	AGGACGG	ACG	1.13	0.11
89	YBR069C	TAT1	-66	AAGCUGU	CUG	1.13	3.83
90	YBR069C	TAT1	-57	UAUAAGG	AAG	0.64	3.55
91	YDR046C	BAP3	-38	AGUAAGA	AAG	0.75	2.24
92	YDR072C	IPT1	-77	UAACUGU	CUG	2.65	2.7
93	YDR293C	SSD1	-236	CAGCUGG	CUG	0.24	3.74
94	YDR298C	ATP5	-70	GCAUUGA	UUG	1.31	2.68
95	YER107C	GLE2	-120	GAAUUGG	UUG	1.78	3.42
96	YGL032C	AGA2	-89	UUGACGU	ACG	0.57	2.3

97	YGR090W	UTP22	-190	AGGACGU	ACG	2.11	2.53
98	YGR138C	TPO2	-109	UCAAAGC	AAG	1.14	2.53
99	YKL064W	MNR2	-92	AAGUUGU	UUG	0.49	2.7
100	YKR003W	OSH6	-29	UUAUUGA	UUG	1.26	2.4
101	YLR286C	CTS1	-187	UUCAUAA	AUA	0.88	2.7
102	YLR286C	CTS1	-181	ACAUUGA	UUG	0.92	2.77
103	YLR430W	SEN1	-106	CUUAUCA	AUC	1.14	2.67
104	YMR238W	DFG5	-93	ACAUUGU	UUG	0.58	2.91
105	YPL020C	ULP1	-71	AAAAUUA	AUU	0.86	3.04
106	YDR387C	CIN10	-79	UCCAUGG	AUG	1.08	2.1
107	YEL016C	NPP2	-96	UAUAUGA	AUG	1.03	2.36
108	YEL023C	YEL023C	-465	UAAAUGU	AUG	2.02	3.16
109	YGL032C	AGA2	-108	GAUAUGU	AUG	0.47	3.29
110	YKL053C-A	MDM35	-75	UAUAUGC	AUG	0.61	2.69
111	YOR307C	SLY41	-100	UAUAUGC	AUG	0.5	2.5
112	YPL148C	PPT2	-44	CUAAUGA	AUG	0.78	2.45

Sr. No. 1-20: uORFs repressed at 20 °C, Sr. No. 21-36: uORFs activated at 20 °C

Sr. No. 37-88: uORFs repressed at 37 °C, Sr. No. 89-112: uORFs activated at 37 °C

Supplementary Table 4. List of putative N-terminal extensions identified in this study.

Sr. No.	Gene name	Standard name	Position from mAUG	Context of start codon	Start codon	ΔTE_{NTE} 20 °C	ΔTE_{NTE} 37 °C
1	YAR015W	ADE1	-63	ACUAUUA	AUU	1.06	2.03
2	YBL026W	LSM2	-57	UCAAUAA	AUA	0.8	1.58
3	YBL026W	LSM2	-30	CACAUAU	AUA	0.79	1.71
4	YBL041W	PRE7	-24	CUAUUGA	UUG	1.13	3.06
5	YBR016W	YBR016W	-21	AAGAUAG	AUA	0.02	0.87
6	YBR029C	CDS1	-24	UAACUGU	CUG	1.06	1.17
7	YBR121C	GRS1	-69	AAAUUGU	UUG	1.01	2.22*
8	YBR125C	PTC4	-36	GCCAUUU	AUU	0.88	0.34
9	YBR172C	SMY2	-60	UCCUUGA	UUG	1.65	0.37
10	YBR188C	NTC20	-48	UACAUCU	AUC	1.09	1.59
11	YBR188C	NTC20	-27	UACUUGU	UUG	0.53	1.25
12	YBR194W	AIM4	-27	AUCAUUU	AUU	1.01	1.73
13	YCL031C	RRP7	-15	CAGAUAG	AUA	0.95	2.46
14	YCL037C	SRO9	-33	AACGUGU	GUG	1.54	1.58
15	YCR065W	HCM1	-66	UUAUUGA	UUG	0.92	0.95
16	YDL046W	NPC2	-33	UAAAUCU	AUC	0.55	0.75
17	YDL084W	SUB2	-21	UUCAUUU	AUU	1.44	0.93
18	YDL141W	BPL1	-108	AAGUUGU	UUG	0.77	1.32
19	YDR032C	PST2	-54	AUAAUAU	AUA	1.31	0.52*
20	YDR032C	PST2	-45	UCAAUAA	AUA	1.31	0.58
21	YDR077W	SED1	-24	AAAAUAA	AUA	1.54*	0.49*
22	YDR086C	SSS1	-30	AAGAUAA	AUA	1.72	0.86
23	YDR097C	MSH6	-45	UAAUUGG	UUG	2.51	2.23
24	YDR144C	MKC7	-42	CGCUUGA	UUG	0.27	2.52
25	YDR245W	MNN10	-27	GGAAUAA	AUA	0.49	1.7
26	YDR245W	MNN10	-24	AUAAUUG	AUU	0.49	1.7
27	YDR298C	ATP5	-21	AUUAUUU	AUU	0.98	0.47

28	YDR298C	ATP5	-63	GAUAUUU	AUU	1.08	1.27
29	YDR377W	ATP17	-60	AAAUUGU	UUG	0.7	0.81
30	YDR399W	HPT1	-39	UAUAUUA	AUU	0.49	1.78
31	YDR490C	PKH1	-36	CACGUGU	GUG	1.13	0.98
32	YDR514C	YDR514C	-45	GUAAUUU	AUU	0.24	0.63
33	YDR524C-B	YDR524C-B	-42	AUCAUUA	AUU	1.02	0.3*
34	YER019C-A	SBH2	-30	CAGGUGG	GUG	0.78	0.48
35	YER036C	ARB1	-15	AACUUGA	UUG	1.42	0.41
36	YER048W-A	ISD11	-27	AACUUGG	UUG	1.11	1.04
37	YER050C	RSM18	-36	AAGAUGA	AUG	0.46*	1.4
38	YER050C	RSM18	-45	N/A	AUG	0.49*	1.44
39	YER059W	PCL6	-45	GUAUUGA	UUG	0.97	0.46
40	YER091C	MET6	-24	AAUAUAA	AUA	2.23*	0.15*
41	YER112W	LSM4	-33	GAACUGA	CUG	1.73	0.24
42	YFL005W	SEC4	-39	CGUAUCG	AUC	1.49	0.49
43	YFL034C-A	RPL22b	-24	UAUAUCG	AUC	0.83	2.38
44	YFL037W	TUB2	-48	GACAUAG	AUA	0.8	0.74
45	YFL039C	ACT1	-42	UUACUGC	CUG	1.26	0.86
46	YFR039C	OSW7	-66	AUCAUAU	AUA	0.97	1.08
47	YGL003C	CDH1	-81	GACUUGG	UUG	0.42	1.89
48	YGL003C	CDH1	-45	CCGAUUU	AUU	0.41	2.58
49	YGL037C	PNC1	-30	UCUUUGU	UUG	0.6	0.92
50	YGL160W	AIM14	-54	UACAUUU	AUU	1.44	0.96
51	YGL179C	TOS3	-51	GCCUUGA	UUG	0.59	3.76
52	YGL215W	CLG1	-69	AUUAUUA	AUU	0.86	0.53
53	YGR078C	PAC10	-21	UAUAUAA	AUA	0.02	1.75
54	YGR146C	ECL1	-69	AGAAUAA	AUA	1.67	1.49
55	YGR146C	ECL1	-66	AUAAUUA	AUU	1.67	1.49
56	YGR250C	RIE1	-87	ACCCUGA	CUG	1.73	1.1
57	YGR281W	YOR1	-15	CCGUUGC	UUG	0.46	0.01*
58	YHL034C	SBP1	-33	CAAAAGA	AAG	1.41	0.88
59	YHR064C	SSZ1	-24	ACAAUCA	AUC	1.19	1.44

60	YHR089C	GAR1	-30	N/A	CUG	1.2	0.4
61	YHR162W	MPC2	-36	UAAACGA	ACG	1.65	0.56
62	YHR179W	OYE2	-24	AAUAUAG	AUA	0.67	0.96
63	YHR183W	GND1	-42	GCCAUUA	AUU	0.88	0.58
64	YHR206W	SKN7	-54	UCUUUGU	UUG	1.29	2.63
65	YHR216W	IMD2	-24	UUAAUAA	AUA	1.07	0.03
66	YIL051C	MMF1	-24	CACAUAC	AUA	1.78	0.81
67	YIL083C	CAB2	-48	AAACUGU	CUG	0.38	0.21
68	YIL124W	AYR1	-21	AUAUUGA	UUG	1.08	0.85
69	YJL020C	BBC1	-45	CAACUGC	CUG	1.29	0.49
70	YJR077C	MIR1	-21	AAGAUCA	AUC	0.07	0.08
71	YKL035W	UGP1	-18	CAAGUGU	GUG	0.95	1.12
72	YKL104C	GFA1	-24	CAAAUCA	AUC	1.56	0.85
73	YKL112W	ABF1	-81	CAACUGC	CUG	0.91	0.64
74	YKL171W	NNK1	-39	CUAACGA	ACG	1.15	0.78
75	YKR068C	BET3	-24	UCAUUGA	UUG	0.43	0.65
76	YKR079C	TRZ1	-42	AGAACGA	ACG	2.33	2.09
77	YKR079C	TRZ1	-162	AAACUGU	CUG	1.08	1.6
78	YKR079C	TRZ1	-129	CACUUGA	UUG	1.1	1.65
79	YLL024C	SSA2	-24	CAGAUUU	AUU	0.73	0.32
80	YLR006C	SSK1	-36	UAAAUCA	AUC	0.96	1.11
81	YLR008C	PAM18	-36	UUAAUAA	AUA	1.29	0.6
82	YLR008C	PAM18	-30	AGGUUGC	UUG	0.15	0.47
83	YLR047C	FRE8	-72	CAGAUUA	AUU	1.41	0.15
84	YLR110C	CCW12	-24	GUCAUUC	AUU	1.06	0.75
85	YLR259C	HSP60	-21	AUCAUAA	AUA	1.11	0.79
86	YLR276C	DBP9	-21	AAACUGC	CUG	2.84	1.61
87	YLR340W	RPP0	-18	AAUACGU	ACG	1.45	0.53*
88	YLR362W	STE11	-30	AGUAUGA	AUG	0.45	1.89
89	YML038C	YMD8	-54	CACUUGA	UUG	0.54	0.45
90	YML038C	YMD8	-48	AAGUUGU	UUG	0.67	0.42
91	YML038C	YMD8	-36	CGAGUGU	GUG	0.73	0.4

92	YML080W	DUS1	-69	AAAGUGC	GUG	1.23	1.54
93	YMR080C	NAM7	-39	CGAAUAU	AUA	0.69	0.45
94	YMR088C	VBA1	-87	GCGAUUU	AUU	0.65	1.77*
95	YMR088C	VBA1	-66	AUUUUGU	UUG	0.55	2.57*
96	YMR088C	VBA1	-48	UCUUUGU	UUG	0.62	2.37*
97	YMR108W	ILV2	-66	AGAAAGU	AAG	1.04	0.8
98	YMR108W	ILV2	-105	CAUUUGA	UUG	1.08	0.58
99	YMR207C	HFA1	-381	ACCAUAU	AUA	0.88	1.45
100	YMR207C	HFA1	-372	ACAAUUA	AUU	0.88	1.46
101	YMR297W	PRC1	-39	GAGAUUG	AUU	0.95	1.14
102	YNL064C	YDJ1	-42	AAACUGA	CUG	0.72	0.9
103	YNL139C	THO2	-39	CAGUUGA	UUG	1.17	0.75
104	YNL189W	SRP1	-93	UAAUUGA	UUG	1.29	1.23
105	YNL200C	NNR1	-57	CAUAUUU	AUU	1.29	1.08
106	YNL216W	RAP1	-54	CCGUUGU	UUG	1.73	0.38
107	YNL216W	RAP1	-33	UACAUAA	AUA	1.4	0.48
108	YNL219C	ALG9	-51	UAGCUGU	CUG	1.16	0.98
109	YNR001C	CIT1	-18	AAUAUAA	AUA	0.99	0.7
110	YNR028W	CPR8	-30	AAGAUUA	AUU	1.73	1.14
111	YOL086C	ADH1	-24	AGCAUAC	AUA	1.2	0.6
112	YOL086C	ADH1	-33	GCUAUAC	AUA	1.08	0.53*
113	YOL136C	PFK27	-66	AAUAUUU	AUU	1.06	0.33
114	YOL136C	PFK27	-57	GAUAUAA	AUA	1.1	0.14
115	YOL155C	HPF1	-36	UCGUUGA	UUG	1.24	1.4
116	YOR049C	RSB1	-84	AAAAUGU	AUG	0.88	2.44
117	YOR065W	CYT1	-90	GUUCUGA	CUG	1.92	2.05
118	YOR085W	OST3	-21	CGCAUCA	AUC	1.47	0.74
119	YOR086C	TCB1	-18	CAAUUGC	UUG	0.16*	0.99
120	YOR089C	VPS21	-24	AAAAUAU	AUA	1.66	0.05
121	YOR198C	BFR1	-27	AGCAUAU	AUA	0.79	0.57
122	YOR209C	NPT1	-30	CUUUUGU	UUG	1.64	1.01
123	YOR335C	ALA1	-75	AAGACGA	ACG	0.87	0.81

124	YOR335C	ALA1	-48	AACUUGA	UUG	1.24	1.05
125	YOR342C	YOR342C	-60	UACAUUA	AUU	1.2	1.85
126	YPL117C	IDI1	-48	UAUUUGG	UUG	0.66	0.47
127	YPL240C	HSP82	-42	AAAAUAG	AUA	1.11	0.26*
128	YPR036W	VMA13	-18	GCACUGG	CUG	0.74	0.25
129	YPR181C	SEC23	-45	CCAGUGU	GUG	0.74	0.65
130	YPR181C	SEC23	-54	UAACUGA	CUG	0.62	0.7

ΔTE_{NTE} with FDR < 0.1 are marked with *.

Supplementary Table 5. List of datasets generated in this study and their GEO accession numbers.

Sr. No.	Strain	Relevant genotype	Growth conditions	Growth temperature	Source	Description	GEO accession number
1	BY4741	WT	SC-Ura	20 °C	This study	RNA-Seq replicate 1	GSM4065752
2	BY4741	WT	SC-Ura	20 °C	This study	RNA-Seq replicate 2	GSM4065753
3	BY4741	WT	SC-Ura	30 °C	This study	RNA-Seq replicate 1	GSM4065754
4	BY4741	WT	SC-Ura	30 °C	This study	RNA-Seq replicate 2	GSM4065755
5	BY4741	WT	SC-Ura	37 °C	This study	RNA-Seq replicate 1	GSM4065756
6	BY4741	WT	SC-Ura	37 °C	This study	RNA-Seq replicate 2	GSM4065757
7	BY4741	WT	SC-Ura	20 °C	[19]	Ribo-Seq replicate 1	GSM2895484
8	BY4741	WT	SC-Ura	20 °C	[19]	Ribo-Seq replicate 2	GSM2895485
9	BY4741	WT	SC-Ura	30 °C	[19]	Ribo-Seq replicate 1	GSM2895488
10	BY4741	WT	SC-Ura	30 °C	[19]	Ribo-Seq replicate 2	GSM2895489
11	BY4741	WT	SC-Ura	37 °C	[19]	Ribo-Seq replicate 1	GSM2895490
12	BY4741	WT	SC-Ura	37 °C	[19]	Ribo-Seq replicate 2	GSM2895491
