# Supplementary material for: Temperature-dependent regulation of upstream open reading frame translation in S. cerevisiae
Source: BMC Biol. 2019 Dec 6;17:101. doi: 10.1186/s12915-019-0718-5 (PMC6898956; doi:10.1186/s12915-019-0718-5)
Supplement: Supplementary file 4 — Additional file 4. Supplementary Methods [68]. [file 12915_2019_718_MOESM4_ESM.docx]

**Supplementary Methods**

**The 5’-UTRs of mRNAs with uORFs tend to be longer and more structured than the genomic average.**

To determine whether the general characteristics of the 5’-UTRs of mRNAs with uORFs are different than those of mRNAs that don’t have evidence of translated uORFs, we examined their lengths and propensities to form secondary structures. We interrogated the PARS dataset of ~2700 yeast mRNA 5’-UTR lengths and propensities of forming secondary structures [40]. As described previously, we identified 1367 uORFs (NCC and AUG, Figure 3A ) which were present on mRNAs (*uORF mRNAs*) with an average 5’-UTR length of 195 nt, which was significantly greater than the average 5’-UTR length of ~79 nt calculated for all mRNAs (*All mRNAs*) in the PARS dataset (Additional file 1: Figure S6A). We also examined the mRNAs that do not show evidence of an actively translated uORF (*Non-uORF mRNAs*) as identified by our pipeline discussed in Figure 3A. These 2157 non-uORF mRNAs had an average 5’-UTR length of 66 nt, significantly shorter than the average for *All mRNAs*. Figure S6A (Additional file 1) shows the cumulative fraction distribution of these three sets of mRNAs.

To examine 5’-UTR secondary structure, we analyzed the PARS (Parallel Analysis of RNA Structure) data available from the same study [40]. In this study, each nucleotide in ~3000 mRNAs was assigned a PARS score, which is a measure of its propensity to be in the double stranded conformation. The PARS score is based on the susceptibility of each nucleotide in the transcribed mRNAs to in vitro digestion with RNase V1 (specific to double-stranded mRNA) and RNase S1 (specific to single-stranded mRNA). Higher PARS score indicates a greater tendency of a nucleotide to be in a double-stranded conformation. We analyzed the PARS scores for each of the sets of mRNAs: *All mRNAs, uORF mRNAs and non-uORF mRNAs.* We considered several PARS features, each of which is an indication of an extent to which a region of the 5’-UTR or coding region near the 5’-UTR can form a secondary structure (Additional file 1: Figure S6B, adapted from [68]). These features include sum of PARS scores of all the nucleotides (nt) present in the 5’-UTR (*Total*); average PARS score per nt (*Average*); sum of PARS scores for the first 30 nts (*First30*); sum of PARS scores for 30 nts surrounding the start codon (for mRNAs with a 5’-UTR of ≥15 nt; *Start30*); and highest total PARS score measured for a 30-nt region anywhere across the 5’-UTR (*Max30*). We also analyzed PARS scores downstream from the mAUG (start codon of the main ORF), with an interval of +1 to +30 (*Plus15*).

Interestingly, the *uORF mRNAs* were shown to have higher PARS scores than *All mRNAs* for most of the PARS features considered (Additional file 1: Figure S6C, compare red versus purple columns). Notably, the *Non-uORF mRNAs* showed significantly lower PARS scores for some of the PARS features compared to *All mRNAs* (Additional file 1: Figure S6C, compare red versus blue columns), suggesting that these *non-uORF mRNAs* are less structured than the genomic average. Together, these data indicate that mRNAs containing actively-translated uORFs typically have longer and more structured 5’-UTRs than those without uORFs.
